# Treatment with the Immunomodulator AIC649 in Combination with Entecavir Produces Antiviral Efficacy in the Woodchuck Model of Chronic Hepatitis B

**DOI:** 10.3390/v13040648

**Published:** 2021-04-09

**Authors:** Kyle E. Korolowicz, Manasa Suresh, Bin Li, Xu Huang, Changsuek Yon, Xuebing Leng, Bhaskar V. Kallakury, Robin D. Tucker, Stephan Menne

**Affiliations:** 1 Department of Microbiology & Immunology, Georgetown University Medical Center, Washington, DC 20057, USA; kek89@georgetown.edu (K.E.K.); ms3687@georgetown.edu (M.S.); Bin.Li@georgetown.edu (B.L.); xh61@georgetown.edu (X.H.); cy33@georgetown.edu (C.Y.); xl318@georgetown.edu (X.L.); 2 Department of Pathology, Georgetown University Medical Center, Washington, DC 20057, USA; kallakub@georgetown.edu; 3 Division of Comparative Medicine, Georgetown University Medical Center, Washington, DC 20057, USA; Robin.Tucker@georgetown.edu

**Keywords:** *parapoxvirus*, TLR9, entecavir, woodchuck, chronic hepatitis B, antiviral efficacy, immune response

## Abstract

As current interventions for chronic hepatitis B (CHB) rarely induce cure, more effective drugs are needed. Short-term treatment of woodchucks with the novel immunomodulator AIC649, a parapoxvirus-based stimulator of toll-like receptor 9 dependent and independent pathways, has been shown to reduce viral DNA and surface antigen via a unique, biphasic response pattern. The present study evaluated long-term AIC649 treatment in combination with Entecavir for potency and safety in woodchucks. AIC649 monotreatment induced modest reductions in serum viral DNA and surface and e antigens that were associated with the same biphasic response pattern previously observed. Entecavir monotreatment reduced transiently viremia but not antigenemia, while AIC649/Entecavir combination treatment mediated superior viral control. Undetectability of viral antigens and elicitation of antibodies in AIC649/Entecavir-treated woodchucks correlated with the expression of interferons and suppression of viral replication in liver. Combination treatment was well tolerated, and liver enzyme elevations were minor and transient. It was concluded that the AIC649-mediated effects were most likely based on an improvement and/or reconstitution of antiviral immune responses that are typically deficient in CHB. As a combination partner to Entecavir, the antiviral efficacy of AIC649 was markedly enhanced. This preclinical study supports future evaluation of AIC649 for treatment of human CHB.

## 1. Introduction

Chronic infection with the hepatitis B virus (HBV) is a major public health concern. It is estimated that 257 million individuals worldwide are chronic carriers of HBV who have an elevated risk of developing chronic hepatitis B (CHB), liver cirrhosis, and hepatocellular carcinoma (HCC) [1]. Approximately 887,000 individuals die each year due to HBV-associated liver disease progression [1]. While effective therapeutic interventions exist for CHB, HBV-infected patients often require prolonged or even lifelong treatment. Several direct-acting antivirals are approved for therapy of CHB, but these nucleos(t)ide analogs (NAs) are rarely curative as they do not affect the viral covalently-closed circular (ccc) DNA genome within the nucleus of infected hepatocytes [2]. This molecule functions as a template for transcription of the pre-genomic (pg) RNA needed for HBV replication and of several RNAs encoding viral proteins [3]. Use of NAs is further hampered by the emergence of drug resistant variants during treatment, the risk of relapse upon treatment discontinuation, and unwanted side effects [4]. Drugs that target directly HBV cccDNA are currently not available for use in patients. Indirect evidence for treatment-induced reduction of this viral molecule includes the loss of HBV surface antigen (HBsAg), but even after five years of therapy with current NAs, induction of a functional cure, which is defined as clearance of circulating HBV DNA and HBsAg with or without subsequent seroconversion to antibodies against HBsAg (anti-HBs antibodies) [5], is a rare event and only achieved in 3–4% of patients on average [6,7]. Since HBV persistence is thought to be the consequence of a functionally impaired immune response due to the high loads of tolerizing viral proteins in the periphery and liver [8,9], immunomodulators are also applied for HBV therapy. However, prolonged use of interferon-alpha (IFN-α) and alternate formulations (e.g., pegylated IFN-α) is again restricted because of treatment-limiting adverse effects [5,10]. In addition, variability in the treatment outcome is a common observation with IFN-α, when provided as a single agent or in combination with NAs, and functional cure is still limited to less than 10% of patients on average after two years of therapy [6,7,11]. Therefore, one major goal to be achieved by new HBV therapeutics is to mimic the benefits of IFN-α therapy in regard to inducing a functional cure but in more than one-tenth of HBV-infected patients after a finite course of treatment, and without the side effects associated with the systemic administration of this pleiotropic cytokine.

One area of focus for treatment of CHB via immunomodulation is the development of agonists targeting pathogen recognition receptors, such as retinoic acid-inducible gene I (RIG-I) and toll-like receptors (TLRs). The latter involve mainly TLR7, 8, and 9 that are highly expressed in dendritic cells (DCs), B lymphocytes, macrophages, and/or other antigen presenting cells (APCs) and are located on endosomal membranes where they usually sense either single-stranded or double-stranded viral RNA or double-stranded unmethylated viral DNA containing CpG motifs [12]. Agonistic activation of these receptors with small molecules can lead to the induction of robust antiviral immune responses [12,13,14] that are thought to be crucial for resolution of HBV infection [8,9]. Several of these agonists targeting RIG-I (SB 9200), TLR7 (GS-9620, APR002, and RG7854), TLR8 (GS-9688), and TLR9 (CpG oligonucleotides), alone or in combination with NAs, have been successfully tested in the woodchuck animal model of CHB [15]. Clinical trials in patients with CHB were performed thereafter or are ongoing for some of these compounds [15,16,17,18,19,20,21]. TLR7 agonism for up to 12 weeks with GS-9620 did not mediate antiviral efficacy at tolerated doses in patients either untreated or treated with NAs [16,17,22], but induced IFN-stimulated genes without serum IFN [23] and augmented T- and natural killer- (NK) cell responses in blood [18]. TLR8 stimulation for 24 weeks by GS-9688 at two separate doses in patients undergoing NA treatment was well-tolerated, induced a dose-dependent IL-12 response in blood, and resulted in modest but durable declines in HBsAg, with 5 and 16% of patients achieving loss of HBsAg or HBV e antigen (HBeAg), respectively [20]. RIG-I agonism for 12 weeks with SB 9200 at accelerating doses in NA-naïve patients mediated dose-dependent reductions in HBV DNA, and 22% of patients achieved dose-independent declines in HBsAg of >0.5 log_10_ either at the end of RIG-I stimulation or following the switch to 12 weeks of NA treatment [21]. However, when SB 9200 at a higher dose was administered for 24 weeks to patients undergoing NA treatment, hepatic adverse events occurred and one patient developed a progressive liver failure and died from multi-organ failure, leading to the immediate termination of all trials with this immunomodulator [24]. It is of note that the latter treatment regimen was not tested for safety and antiviral efficacy in preclinical animal models.

The Eastern woodchuck (*Marmota monax*) is naturally infected with the woodchuck hepatitis virus (WHV), a member of the genus *Orthohepadnavirus* that is closely related to HBV regarding its genome structure, virion morphology, and mechanisms of infection and replication [25,26,27]. Comparable to HBV, WHV causes age-dependent acute and chronic outcomes of infection, and experimental infection of neonatal woodchucks resembles the vertical transmission route of HBV in humans [25,28,29]. Immunopathogenesis and liver disease progression to HCC mediated by WHV in woodchucks parallels HBV infection in humans more so than in any other animal model currently available for HBV research [25,28,30,31,32,33,34,35]. Furthermore, woodchucks are fully immunocompetent and the animal model is supported by the recent identification of the liver transcriptome and genome [36,37]. Thus, woodchucks are increasingly utilized for the evaluation of new antiviral drugs for treatment of CHB and HCC [28,31,33,38,39,40,41,42,43,44,45], and their preclinical use is predictive of therapeutic efficacy of NAs [40,46] and immunomodulators [15,47,48] against HBV in patients.

APCs, such as conventional (c) and plasmacytoid (p) DCs, prime T- and B-cells for generating an effective immune response against HBV [49]. These APCs, however, are functionally impaired in patients with CHB, resulting in insufficient induction of adaptive immune responses that are unable to control persistent infection with HBV. For example, lower numbers of blood pDCs with reduced TLR9 expression are present in HBV-infected patients when compared to uninfected individuals [50]. Suppression of interleukin (IL) 12 and IFN-α and upregulation of IL-10 production in response to various maturation stimuli have been noted in cDCs and pDCs isolated from these patients, and these cells were less able to stimulate T-cell activation and proliferation [51,52]. Thus, enhancing the priming of T- and B-cell responses by APCs appears a reasonable strategy for inducing a functional cure of CHB, and a reversal of the functional impairment of pDCs may be possible via TLR9 agonism. Compared to TLR7 stimulators, TLR9 agonists, such as CpG-containing synthetic oligonucleotides, induce a slower but more sustained and broader type I IFN expression in human blood cells, and the kinetic, quantity, and quality of IFN-α and IFN-β produced translates into different patterns of IFN-stimulated gene (ISG) induction (e.g., higher expression of chemokines, transcription factors, proteins involved in apoptosis, and major histocompatibility complex class [MHC] II) [53]. In addition, TLR9 stimulation of B-cells with CpG oligonucleotides enhances their differentiation into immunoglobulin-secreting plasma cells [14].

Another TLR9 stimulator is AIC649, an inactivated parapoxvirus ovis (PPVO) particle preparation under development by AiCuris (Anti-infective Cures GmbH, Wuppertal, Germany) for the treatment of human CHB. Since PPVO has a CG-rich double-stranded DNA genome, TLR9 in murine pDCs is the main receptor sensing AIC649 following complement opsonization and cellular uptake of viral particles [54,55] (reviewed in [56]). Stimulation of TLR9 but not of TLR2 and TLR4 then elicits and releases type I IFNs via the myeloid differentiation primary response gene 88 (MyD88) pathway [54,55]. However, AIC649 also stimulates additional TLR-independent pathways. In murine cDCs, if PPVO particles localize in the cytosol instead of endosomal compartments after cellular uptake, they may stimulate DNA-sensing receptors, most likely DNA-dependent activator of IFN regulatory factors (DAI) for the production of type I IFNs [54,55]. Furthermore, AIC649 activates human monocytes and pDCs through signaling via CD14 [57]. Moreover, the immunostimulatory activity of AIC649 in human immune cells and in mice is associated with PPVO particles [57,58] and depends at least on two viral proteins [59].

In addition to type I IFNs, AIC649 stimulation of human immune cells mediates tumor necrosis factor-alpha (TNF-α) and IL-12 secretion directly, and also IFN-γ expression in NK- and/or pre-activated T-cells [58]. Furthermore, in human blood cells and in mice, AIC649 induces an autoregulated loop of initial upregulated inflammatory (TNF-α, IL-6 and IL-8) and T helper cell type I (Th1) related cytokines (IFN-α, INF-γ, IL-12, and IL-18), which activates innate (mainly DCs and NK-cells) and adaptive immune responses (Th1 cells and cytotoxic T lymphocytes [CTLs]) [56,58]. The inflammatory response, however, is limited by subsequent upregulation of anti-inflammatory and Th2 type cytokines [56,58]. This interplay of innate and adaptive immune responses induced by AIC649 then leads to a concerted antiviral response against chronic infections with unrelated viruses in several animal models. For example, AIC649 protected mice from lethal herpes simplex virus type 1 infection [58,59,60]. AIC649 also shielded guinea pigs from recurrent genital herpes disease [58] and reduced herpes lesions and viral shedding in this animal model [61]. AIC649 further suppressed viral replication in an in vitro hepatitis C virus (HCV) replication model [62] and in HBV transgenic mice [58,62,63]. In the latter animal model, twice-weekly peritoneal AIC649 administration for four weeks reduced the viral load by approximately 2.0 log_10_ that was comparable to the antiviral effect achieved by twice-daily oral (po) dosing with the nucleotide analog Tenofovir [63]. In woodchucks with CHB, AIC649 appears less potent when administered intramuscularly (im), because twice-weekly treatment for eight weeks reduced serum viremia and antigenemia by less than 1.0 or 0.4 log_10_, respectively [63]. The previous woodchuck study, however, revealed a biphasic treatment response pattern that was characterized by an initial increase in viral DNA and surface antigen followed by a modest but durable decline in both molecules.

In the present woodchuck study, the antiviral efficacy of AIC649 was evaluated using an altered treatment regimen consisting of an initial intravenous (iv) administration for active treatment and a subsequent im administration for treatment maintenance, as well as longer treatment durations. AIC649 administration by iv injection for twelve weeks was selected for active treatment in anticipation that a more targeted delivery of the immunomodulator into the liver and a longer-lasting stimulation of TLR9-dependent and TLR-independent pathways in intrahepatic APCs would result in a greater antiviral efficacy when previously achieved. A switch to im administration of AIC649 was selected for the nine weeks of maintenance treatment in anticipation that the antiviral effect already induced would be further enhanced or at least preserved. This switch also considered an expected decline in patient compliance with prolonged, twice-weekly iv treatment during future clinical testing of AIC649. Since most therapeutic interventions based on immunomodulation will likely be provided in parallel or as an add-on to standard of care antiviral treatment with NAs, AIC649 was further assessed in combination with the nucleoside analog Entecavir (ETV) to more accurately reflect the intended clinical use of the immunomodulator. Thus, the overall hypothesis tested was that active and maintenance treatment of woodchucks with AIC649, alone and together with ETV, would result in antiviral efficacy against WHV via improved or reconstituted innate and adaptive immune responses and that the antiviral effect would be greater than previously achieved during monotreatment [63].

## 2. Materials and Methods

### 2.1. Investigational Drugs

AIC649 was manufactured by IDT Biologika (Dessau-Roßlau, Germany), provided as a lyophilizate, and reconstituted in pyrogen-free water for injection. AIC649 was administered iv or im at a volume of 1.0 mL that equaled to a dose of 1 × 10^9^ chemically inactivated PPVO particles of strain NZ-2 per animal. Vehicle AIC (IDT Biologika) was also provided as a lyophilizate, reconstituted in pyrogen-free water, and injected iv or im at a volume of 1.0 mL per animal. ETV was purchased from Selleck Chemicals (Munich, Germany). ETV powder was dissolved in a sterile, isotonic saline solution to obtain a concentration of 0.2 mg/mL. The solution was then administered orally (po) at a volume of 1.0 mL per kg of body weight in woodchuck liquid diet (Dyets, Bethlehem, PA, USA) that equaled to a dose of 0.2 mg/kg of body weight. Vehicle ETV was a sterile isotonic saline solution and administered po at a volume of 1.0 mL per kg of body weight in woodchuck liquid diet (Dyets).

### 2.2. Animals

All 20 woodchucks were inoculated at 3 days of age with strain 7 of WHV (WHV7) [29] and maintained at the animal facilities of Northeastern Wildlife, Inc. (Harris, ID, USA) until an age of 15 to 17 months. Following transfer to the animal facilities at Georgetown University, woodchucks were confirmed as established chronic WHV carriers based on the presence of WHV DNA, WHsAg, and WHeAg and absence of antibodies against WHsAg and WHeAg in serum (anti-WHs and anti-WHe antibodies). The HCC-free status of all animals was confirmed by ultrasonography. Most animals presented with low serum gamma-glutamyl transferase (GGT) activity, an oncogenic marker in woodchucks [64], while three animals had slightly to moderately elevated liver enzyme levels.

Woodchucks were allocated to four groups and randomized within blocks and factors. The block for stratification was sex. The factor was the categorial variable body weight (low, medium, and high). If needed, animals were moved between the groups based on other parameters, including pretreatment serum viral markers (WHV DNA, WHsAg, and WHeAg), and serum liver enzyme levels (GGT, sorbitol dehydrogenase (SDH), aspartate aminotransferase (AST) and alanine aminotransferase (ALT)), for achieving comparable ranges within each group. Hematology parameters were not included but checked to ensure comparable ranges. Animal procedures involving Vehicle AIC and AIC649 iv administration, blood collection, and percutaneous liver biopsy were performed under 1–5% isoflurane inhalation and/or ketamine (25.0–50.0 mg/kg)/xylazine (1.0–5.0 mg/kg) im injection anesthesia. Animal research staff was not blinded in regard to treatment administration and animal procedures. However, laboratory research staff was blinded to animal group/treatment allocation during sample processing and analysis.

### 2.3. Study Design

**Figure 1:**
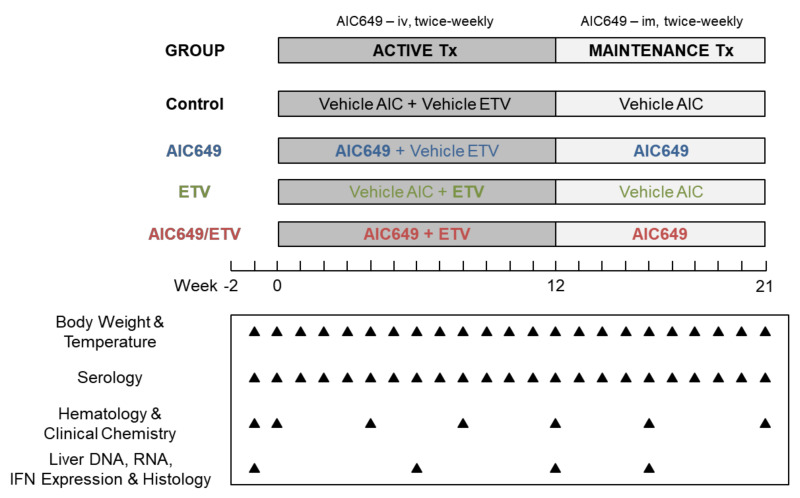
Study design. For active treatment, AIC649 was administered twice-weekly by iv injection during the initial 12 weeks of the study to woodchucks of the AIC649 and AIC649/Entecavir (ETV) Groups. ETV was provided by daily po administration during the initial 12 weeks of the study to woodchucks of the ETV and AIC649/ETV Groups. Following the switch to maintenance treatment, AIC649 was administered twice-weekly by im injection during the next 9 weeks to woodchucks of the AIC649 and AIC649/ETV Groups. Antiviral efficacy and safety of the mono and combination treatment regimens were compared to double placebo treatment of woodchucks of the Control Group, as well as between the AIC649, ETV, and AIC649/ETV Groups. Arrows indicate the time of measurements for the specific parameters listed. Abbreviation: iv, intravenous, im, intramuscular, Tx, treatment, IFN, interferon.

Under the study part supported by the United States National Institutes of Health (NIH), AIC649 at a dose of 1 × 10^9^ inactivated PPVO particles/animal was administered twice-weekly to five woodchucks (AIC649 Group); first iv for 12 weeks during active treatment and then im for 9 weeks during treatment maintenance. These animals also received daily oral Vehicle ETV during the initial 12 weeks (Figure 1). Oral ETV at a dose of 0.2 mg/kg was administered daily to another five woodchucks during the initial 12 weeks (ETV Group). These animals further received Vehicle AIC twice-weekly by iv injection for 12 weeks followed by im injection for 9 weeks. Combination treatment with AIC649 and ETV was administered to another five woodchucks (AIC649/ETV Group) and provided as described above for the single agents. Double placebo treatment was provided to another five woodchucks (Control Group). These animals received twice-weekly Vehicle AIC by iv injection for 12 weeks followed by im injection for 9 weeks and daily oral Vehicle ETV during the initial 12 weeks. The results of the NIH-funded portion of the study are presented below, while the results of the AiCuris-supported study part, involving maintenance treatment in all groups for additional 15 weeks, were reported in a conference abstract [65].

Changes in serum and liver viremia and antigenemia were evaluated for determining antiviral effects of the mono and combination treatment regimens. The primary endpoint included reductions in serum WHV DNA, WHsAg, and WHeAg at the end of active treatment and again at the end of maintenance treatment. Changes in the intrahepatic expression of IFN-α, IFN-β and IFN-γ, as well as the elicitation of anti-WHs and anti-WHe antibodies, were assessed as a secondary endpoint for determining the induction of innate and adaptive immune responses associated with the individual treatment regimens. Clinical observations and changes in body weight, body temperature, hematology, and clinical chemistry, including liver enzymes, were obtained at regular intervals for monitoring drug safety. Mortality associated with AIC649 and/or ETV treatment was not observed. One woodchuck died and four animals underwent scheduled euthanasia during the study. Woodchuck M5044 (AIC649 Group) died from hemorrhage following the liver biopsy procedure at week 12. Woodchucks F5057 (Control Group), M5041, M5045, and F5053 (ETV Group) were euthanized during weeks 21, 4, 14, or 21, respectively, due to the development of large liver tumors/end-stage HCC.

### 2.4. Blood Collection

Blood samples for determining serology, hematology, and clinical chemistry were obtained via femoral venipuncture from anesthetized woodchucks after oral dosing with Vehicle ETV or ETV and always prior to iv or im administration of Vehicle AIC or AIC649 (Figure 1). Blood samples for serology were collected during pretreatment at week −1 and T0, and then weekly during treatment. Blood samples for hematology and clinical chemistry were obtained during pretreatment at week −1 and T0, and then at weeks 4, 8, 12, 16, and 21.

### 2.5. Liver Tissue Collection

Ultrasound-guided, percutaneous liver biopsies for determining WHV nucleic acids, IFN expression, and histology were obtained from anesthetized woodchucks after oral dosing with Vehicle ETV or ETV and always prior to iv or im administration of Vehicle AIC or AIC649 (Figure 1). A disposable liver biopsy 16-gauge needle kit (Bard Biopty/Becton Dickinson, Covington, GA, USA) mounted onto an imaging manifold and connected to an ultrasound instrument and computer (Aloka, Twinsburg, OH, USA) was utilized for obtaining hepatic tissues. The ultrasound probe was applied with sterile imaging gel to the abdominal area of an animal and the resulting hepatic imaging was used to guide the liver punch biopsies. The hollow biopsy needle was inserted through the abdomen into the liver to remove cores of the liver tissue. Post-biopsy, puncture sites were dressed, and the animal was administered analgesia (buprenorphine, 0.01–0.03 mg/kg, subcutaneous) and long-acting benzathine penicillin (10,000–30,000 units/kg, im). Liver tissues were collected during pretreatment at week −1, and then at weeks 6, 12, and 16. Liver samples for analyzing WHV DNA replicative intermediate (RI), WHV cccDNA, and WHV RNA levels, as well as IFN transcript levels, were placed immediately into liquid nitrogen and stored at −80 °C. Liver samples for determining disease progression were stored in phosphate-buffered formalin and subsequently embedded into paraffin.

### 2.6. Serum WHV Parameters

Serum WHV DNA levels were assayed quantitatively by slot-blot hybridization and real-time PCR, as described previously [38] and in the Supplementary Data. The lower limit of detection (LLOD) of the hybridization and PCR assays was 1.0 × 10^7^ or 6.0 × 10^2^ WHV genomic equivalents (ge) or copy numbers per mL serum, respectively. Serum WHsAg levels were assayed quantitatively by an enzyme-linked immunosorbent assay (ELISA) comparable to the assay described previously [66] and in the Supplementary Data. The LLOD of the ELISA was 5.0 ng WHsAg per mL serum. Serum WHeAg levels were assayed qualitatively using a cross-reactive ELISA (DiaSorin, Minneapolis, MN, USA) by following the manufacturer’s protocol. Results were obtained as an optical density read out, and a value of ≤0.097 optical density units (ODU) indicated absence of WHeAg in this study. Serum anti-WHs antibody titers were assayed quantitatively using an established enzyme immunoassays (EIA) as described previously [66] and in the Supplementary Data. The LLOD of the EIA using a 1:100 sample dilution was 100 standard units (StdU) per mL serum. Serum anti-WHe antibody levels were assayed qualitatively using a cross-reactive ELISA (DiaSorin) by following the manufacturer’s protocol. An ODU value of ≥2.286 (i.e., sample ODU value at pretreatment (T0) minus sample ODU value in a given study week) indicated presence of anti-WHe antibodies in this study.

### 2.7. Liver WHV Parameters

Intrahepatic WHV DNA RI levels were assayed quantitatively by Southern blot hybridization, while intrahepatic WHV RNA levels (i.e., pg and surface RNAs) were determined quantitatively by Northern blot hybridization, as described previously [38] and in the Supplementary Data. Woodchuck β-actin was used for the normalization of WHV nucleic acid concentrations. Both hybridization assays provided results spanning up to >1 and >2 orders of magnitude of detection for WHV RNA or WHV DNA RI species, respectively. The LLOD for both assays was 2.0 pg WHV DNA or WHV RNA per µg cellular nucleic acids. WHV cccDNA levels were also quantified directly from the Southern blots. Due to insufficient amounts of remaining liver tissue, a confirmation of the WHV cccDNA results by more sensitive real-time PCR was not possible, and thus this data is not presented.

### 2.8. Hematology and Clinical Chemistry Parameters

Blood samples for hematology and clinical chemistry were analyzed at the Animal Health Diagnostic Center of Cornell University (Ithaca, NY, USA) using parameters validated for woodchucks [64,67]. Hematology parameters included white blood cells, segmented neutrophils, banded neutrophils, lymphocytes, monocytes, eosinophils, basophils, red blood cells, hemoglobin, hematocrit, mean cell volume, mean cell hemoglobin, mean cell hemoglobin concentration, red cell distribution width, platelet count, and mean platelet volume. Clinical chemistry parameters included ALT, AST, SDH, GGT, alkaline phosphatase (ALP), sodium, potassium, chloride, bicarbonate, anion gap, sodium/potassium ratio, urea, creatinine, calcium, phosphate, magnesium, total protein, albumin, globulin, albumin/globulin ratio, glucose, total bilirubin, direct bilirubin, indirect bilirubin, amylase, cholesterol, creatine kinase, iron, total iron binding capacity, percent saturation, lipemia, hemolysis, and icterus.

### 2.9. Liver IFN Expression

Changes in the intrahepatic expression of IFN-α, IFN-β, and IFN-γ were determined by real-time PCR, as described in more detail in the Supplementary Data. In brief, total RNA was isolated from liver tissue using the RNeasy Mini kit (Qiagen, Redwood City, CA, USA) with on-column digestion by RNase-free DNase I (Qiagen). Following reverse transcription of mRNA with the High Capacity cDNA Reverse Transcription kit (Applied Biosystems, Foster City, CA, USA) using oligo(dT), complementary (c) DNA samples were amplified on a QuantStudio 3 Real Time PCR System instrument (Applied Biosystems) with the TaqMan Gene Expression Master mix (Applied Biosystems) and woodchuck-specific primers and probes (Table S1). Woodchuck 18S rRNA expression was used to normalize IFN expression. IFN transcription levels were calculated as a fold-change relative to the pretreatment baseline at week −1 using the formula 2^ΔCt^.

### 2.10. Liver Histology

Paraffin-embedded liver tissues were sectioned (5 micron) and stained with hematoxylin and eosin at the Histopathology & Tissue Shared Resource (HTSR) Laboratory of Georgetown University. Liver samples were then examined by a board-certified pathologist (BVK) under a light microscope. Liver disease progression, including portal and sinusoidal hepatitis, bile duct proliferation, steatosis, fibrosis, and necrosis, was scored by using criteria developed for woodchuck liver [68,69], as well as by using the METAVIR scale for scoring human liver, as described in the Supplementary Data.

### 2.11. Statistical Analysis

All data was inspected for consistency and completeness before statistical analysis. Values below an individual assay LLOD were replaced by the corresponding detection limit (i.e., 600 ge/mL for WHV DNA, 5.0 ng/mL for WHsAg, 100 StdU/mL for anti-WHs antibody, and 2.0 pg WHV DNA or WHV RNA/µg cellular nucleic acids). Data for serum WHV DNA and WHsAg and for liver WHV DNA RI and WHV RNA was transformed to a log_10_ scale and arithmetically averaged prior to statistical analysis. Inter-group statistical comparisons were performed using unpaired Student’s *t*-test with equal variance at each timepoint of the study for changes in the following mean parameters: body weight, body temperature, hematology, clinical chemistry, serum and liver WHV markers, IFN expression, and liver pathology. *p* values < 0.05 were considered statistically significant.

## 3. Results

For assessing the therapeutic efficacy and safety of the immunomodulator AIC649, five woodchucks with established chronic WHV infection each were treated with the following regimens: Double placebo treatment (Control Group), AIC649 monotreatment (AIC649 Group), ETV monotreatment (ETV Group), and AIC649/ETV combination treatment (AIC649/ETV Group) (Figure 1).

### 3.1. AIC649 Modestly Reduced Viremia and Antigenemia in the Periphery, but the Antiviral Efficacy was Enhanced in Combination with ETV

**Figure 2:**
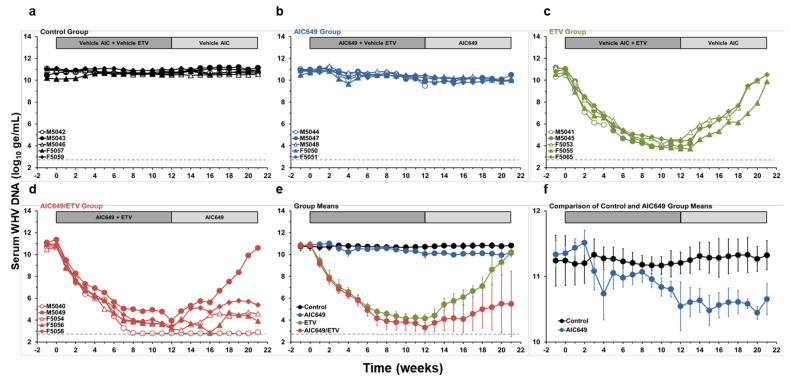
AIC649/ETV combination treatment results in suppression or undetectability of serum viremia in most woodchucks. Changes in serum woodchuck hepatitis virus (WHV) DNA levels relative to pretreatment (week −1 and T0) in woodchucks during (**a**) placebo treatment, (**b**) AIC649 monotreatment, (**c**) ETV monotreatment, (**d**) AIC649/ETV combination treatment, and (**e**) geometric group means. (**f**) The comparison of geometric means of Control and AIC649 Groups illustrates the biphasic change in serum WHV DNA level during initial AIC649 monotreatment. The horizontal dotted line indicates the detection limit for WHV DNA (i.e., 600 ge/mL). The vertical lines represent the standard error of the mean. Viremia levels were significantly reduced compared to the Control Group at week 4 and again during weeks 9–21 in the AIC649 Group (*p* < 0.05) and during weeks 1–21 in both the ETV Group (*p* < 0.05) and the AIC649/ETV Group (*p* < 0.005). Viremia levels were also significantly reduced compared to the AIC649 Group during weeks 1–18 in the ETV Group (*p* < 0.005) and during weeks 1–21 in the AIC649/ETV Group (*p* < 0.05). The viremia level was further significantly reduced compared to the ETV Group at weeks 12, 16, and 19 in the AIC649/ETV Group (*p* < 0.05). Abbreviation: ge/mL, genome equivalents or copy numbers per milliliter.

As expected, serum WHV DNA levels remained unchanged during the study in double placebo treated woodchucks of the Control Group (Figure 2). In animals treated with AIC649 alone, WHV DNA levels initially increased by 0.31 log_10_ on average during the first two weeks of active treatment and then declined transiently by 0.53 log_10_ during the next two weeks, when compared to the Control Group. Additional declines in viremia in the AIC649 Group started at week 9 of active treatment and continued during maintenance treatment (maximum average reduction, 0.91 log_10_ at week 20). As further anticipated, rapid and marked declines in WHV DNA levels were noted in all woodchucks treated with ETV alone (maximum average reduction, 6.69 log_10_ at week 12). After drug withdrawal, all surviving animals in the ETV Group experienced recrudescence of viral replication and WHV DNA levels returned close to the pretreatment baseline. A more pronounced decline in viremia was noted for woodchucks that underwent combination treatment with AIC649 and ETV (maximum average reduction, 7.57 log_10_ at week 12). Following the completion of ETV treatment, WHV DNA levels in woodchucks of the AIC649/ETV Group stayed suppressed or undetectable during maintenance treatment, except for one animal with a viral relapse. Since the WHV DNA levels in the AIC649/ETV Group were significantly different to the ETV Group at weeks 12, 16, and 19, immunomodulation with AIC649 enhanced and extended the antiviral effect mediated by ETV on viremia in most animals.

**Figure 3:**
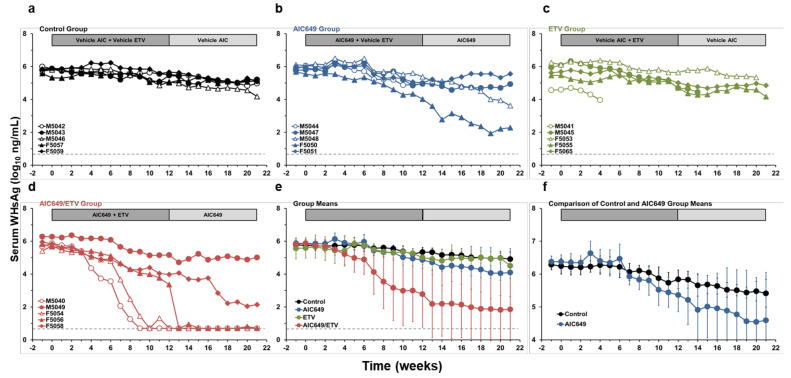
AIC649/ETV combination treatment results in suppression or undetectability of serum surface antigenemia in most woodchucks. Changes in serum WHsAg levels relative to pretreatment (week −1 and T0) in woodchucks during (**a**) placebo treatment, (**b**) AIC649 monotreatment, (**c**) ETV monotreatment, (**d**) AIC649/ETV combination treatment, and (**e**) geometric group means. (**f**) The comparison of geometric means of Control and AIC649 Groups illustrates the biphasic change in serum WHsAg level during initial AIC649 monotreatment. The horizontal dotted line indicates the detection limit for WHsAg (i.e., 5.0 ng/mL). The vertical lines represent the standard error of the mean. Antigenemia levels were significantly reduced compared to the Control Group during weeks 7–21 in the AIC649/ETV Group (*p* < 0.05) but not in the AIC649 and ETV Groups (*p* > 0.05). Antigenemia levels were also significantly reduced compared to the AIC649 Group at weeks 3, 6, 8, and 9 in the AIC649/ETV Group (*p* < 0.05) but not in the ETV Group (*p* > 0.05). The antigenemia level was further significantly reduced compared to the ETV Group at weeks 13 and 14 and during weeks 17–20 in the AIC649/ETV Group (*p* < 0.05).

Placebo treatment and ETV monotherapy had no apparent effect on serum antigenemia, and the WHsAg and WHeAg levels in the Control and ETV Groups were comparable and stayed relatively unchanged during the study (Figure 3 and Figure S1). As noted before for viremia, AIC649 monotreatment also resulted in a 0.41 log_10_ WHsAg increase on average during active treatment, when compared to the Control Group, with peaks observed at weeks 3 and 6. Thereafter, the WHsAg level declined in the AIC649 Group (maximum average reduction, 1.83 log_10_ at week 20) and the reductions were pronounced in two woodchucks during the remainder of active and/or maintenance treatment. A comparable pattern of increase and subsequent decline was further noted for WHeAg in the AIC649 Group. The WHeAg level increased by 0.20 ODU on average at week 3, transiently declined by 0.36 ODU at week 8, and then continued to decline further (maximum average reduction, 0.76 ODU at week 21). The reduction in WHeAg level was also due to mainly two woodchucks with more marked changes during maintenance treatment, and these were the same animals with parallel declines in WHsAg. The AIC649-mediated effect on antigenemia was much more pronounced in combination with ETV, and three woodchucks in the AIC649/ETV Group achieved undetectable WHsAg levels during active and/or maintenance treatment, while the WHsAg load was suppressed in a fourth animal (maximum average reduction, 4.05 log_10_ at week 20). Undetectable or suppressed WHeAg levels were also observed in the same four woodchucks during active and/or maintenance treatment (maximum average reduction, 2.46 ODU at week 21). Since the WHsAg and WHeAg levels in the AIC649/ETV Group were significantly different to the AIC649 Group at weeks 3, 6, 8, and 9 or at weeks 3 and 6 and during weeks 11–21, respectively, the addition of ETV enhanced the antiviral effect produced by immunomodulation with AIC649 on antigenemia in most woodchucks.

### 3.2. AIC649 Modestly Suppressed WHV Replication in the Liver, but the Antiviral Efficacy was Again Enhanced in Combination with ETV

**Figure 4:**
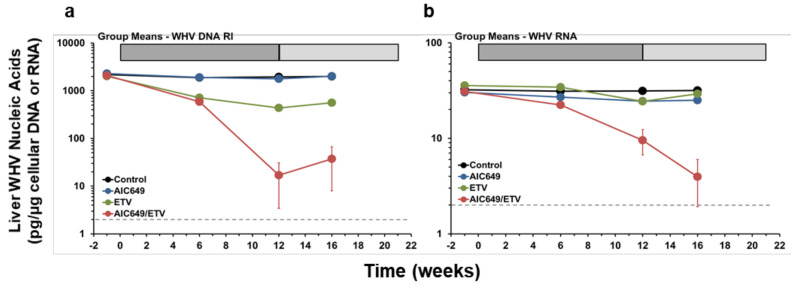
AIC649/ETV combination treatment results in suppression of WHV nucleic acids in the liver. Comparison of geometric means of the Control, AIC649, ETV, and AIC649/ETV Groups relative to pretreatment (week −1) for intrahepatic levels of (**a**) WHV DNA replicative intermediate (RI) and (**b**) WHV RNA. The horizontal dotted line indicates the detection limit for WHV nucleic acids (i.e., 2.0 pg/µg cellular DNA or RNA). The vertical lines represent the standard error of the mean. WHV DNA RI levels were significantly reduced compared to the Control Group at weeks 6, 12, and 16 in both the ETV Group (*p* < 0.01) and the AIC649/ETV Group (*p* < 0.05) but not in the AIC649 Group (*p* > 0.05). WHV DNA RI levels were also significantly reduced compared to the AIC649 Group at weeks 6, 12, and 16 in both the ETV Group (*p* < 0.005) and the AIC649/ETV Group (*p* < 0.05). The WHV DNA RI level was comparable between the ETV and AIC649/ETV Groups (*p* > 0.05). WHV RNA levels were significantly reduced compared to the Control Group at week 16 in the AIC649/ETV Group (*p* < 0.05) but not in the AIC649 and ETV Groups (*p* > 0.05). WHV RNA levels were also significantly reduced compared to the AIC649 Group at week 16 in the AIC649/ETV Group (*p* < 0.005) but not in the ETV Group (*p* > 0.05). The WHV RNA level was further significantly reduced compared to the ETV Group at week 16 in the AIC649/ETV Group (*p* < 0.05).

Correlating with the effects on serum viremia and antigenemia, AIC649 and ETV monotreatment and AIC649/ETV combination treatment suppressed WHV nucleic acids in the liver of woodchucks at varying degrees, while these viral molecules remained nearly unchanged during placebo treatment (Figure 4, Figures S2 and S3). Viral nucleic acid levels in the AIC649 Group were modestly reduced at the end of active treatment and during maintenance treatment (maximum average reduction, week 16, WHV DNA RI, 0.11 log_10_, WHV RNA, 0.09 log_10_). Viral nucleic acids became also reduced in the ETV Group, but the declines were transient and levels relapsed in surviving animals after the end of ETV treatment (maximum average reduction, week 12, WHV DNA RI, 0.66 log_10_, WHV RNA, 0.16 log_10_). The reductions in viral nucleic acids were more pronounced in the AIC649/ETV Group, and the WHV RNA level declined during active and maintenance treatment while the WHV DNA RI level slightly increased after ETV withdrawal at the end of active treatment (maximum average reduction, week 12, WHV DNA RI, 2.09 log_10_; week 16, WHV RNA, 0.89 log_10_). Most woodchucks in this group had markedly suppressed or undetectable viral nucleic acids during treatment and these were essentially the same animals with suppressed or undetectable serum WHV DNA, WHsAg, and WHeAg. Only one animal experienced recrudescence of viral replication during maintenance treatment, and this animal also had a relapse in serum viremia and moderately suppressed antigenemia. The comparison of viral nucleic acids revealed that WHV DNA RI and RNA levels in the AIC649/ETV Group were significantly different to the AIC649 Group at weeks 6, 12, and 16 or at week 16, respectively. In addition, the WHV RNA level in the AIC649/ETV Group was also significantly different to the ETV Group at week 16. This overall indicated that the addition of ETV enhanced the antiviral effect mediated by immunomodulation with AIC649 on WHV replication in liver.

### 3.3. AIC649 Induced Type I and II IFNs in the Liver, but the Intrahepatic IFN Expression was Enhanced in Combination with ETV

**Figure 5:**
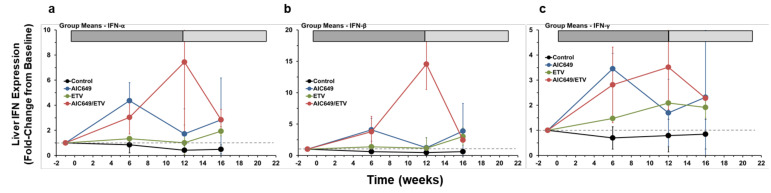
AIC649/ETV combination treatment results in enhanced expression of type I and II interferons (IFNs) in the liver. Comparison of means of the Control, AIC649, ETV, and AIC649/ETV Groups relative to pretreatment (week −1) for intrahepatic transcript levels of (**a**) IFN-α, (**b**) IFN-β, and (**c**) IFN-γ. Fold-change values were calculated relative to the pretreatment baseline, which was set a 1.0 and is indicated by the horizontal dotted line. The vertical lines represent the standard error of the mean. IFN-α transcript levels were significantly increased compared to the Control Group at week 6, at week 16, and at weeks 6, 12, and 16 in the AIC649 Group (*p* < 0.01), ETV Group (*p* < 0.05), or AIC649/ETV Group (*p* < 0.05), respectively (*p* < 0.05). IFN-α transcript levels were comparable between the AIC649, ETV, and AIC649/ETV Groups (*p* > 0.05). Compared to the Control Group, the IFN-β transcript levels were significantly increased at week 6 and at weeks 6, 12, and 16 in the AIC649 Group (*p* < 0.05) or AIC649/ETV Group (*p* < 0.05), respectively, but not in the ETV Group (*p* > 0.05). IFN-β transcript levels were also significantly increased compared to the AIC649 Group at week 12 in the AIC649/ETV Group (*p* < 0.005) but not in the ETV Group (*p* > 0.05). The IFN-β transcript level was further significantly increased compared to the ETV Group at week 12 in the AIC649/ETV Group (*p* < 0.05). IFN-γ transcript levels were significantly increased compared to the Control Group at week 6 and at weeks 6, 12, and 16 in the AIC649 Group (*p* < 0.001) or AIC649/ETV Group (*p* < 0.05), respectively, but not in the ETV Group (*p* > 0.05). IFN-γ transcript levels were comparable between the AIC649, ETV, and AIC649/ETV Groups (*p* > 0.05).

Since stimulation of TLR9-dependent and TLR-independent pathways results in the production of type I IFNs and can lead to the subsequent release of type II IFN by immune cells [12,14,54,55,56,58], the intrahepatic expression of IFN-α, IFN-β, and IFN-γ was analyzed and correlated with the four treatment regimens for determining if immunomodulation with AIC649 induced an innate immune response in woodchucks. Placebo treatment was not associated with elevations in IFN transcription in the Control Group, confirming that the antiviral immune response is deficient in woodchucks with CHB (Figure 5 and Figures S4–S6). Minor and sometimes transient increases in IFN-α, IFN-β, and IFN-γ expression were observed in the ETV Group at or following the end of drug treatment that were mainly attributable to two animals, indicating an immune response of the host to either suppressed or relapsing WHV replication. Although AIC649 monotreatment mediated only modest antiviral effects, it induced pronounced but transient elevations in IFN expression in the AIC649 Group during active treatment. An additional elevation in IFN transcription was noted during maintenance treatment that was due to two animals with increased expression of IFN-α, IFN-β, and IFN-γ. The timing of IFN expression indicated a rapid induction of an immune response in the host by immunomodulation with AIC649. Increases in IFN-α, IFN-β, and IFN-γ expression were also noted during combination treatment in the AIC649/ETV Group, especially at the end of active treatment. Following the switch to maintenance treatment, IFN expression declined but stayed somewhat elevated. The timing of immune response induction was similar to the AIC649 Group, but the magnitude and duration of IFN-α, IFN-β, and IFN-γ expression during active treatment were higher and longer-lasting, and the increase in IFN-β transcript level was significantly different to the AIC649 and ETV Groups at week 12. Importantly, the immune response in the AIC649/ETV Group correlated well with the suppression or undetectability of circulating and intrahepatic WHV markers in most animals. This overall indicated that the addition of ETV enhanced the antiviral innate immune response induced by immunomodulation with AIC649 in the liver.

### 3.4. AIC649 Induced Anti-WHs Antibodies in the Periphery, but Anti-WHe Antibodies were Only Elicited in Combination with ETV

**Figure 6:**
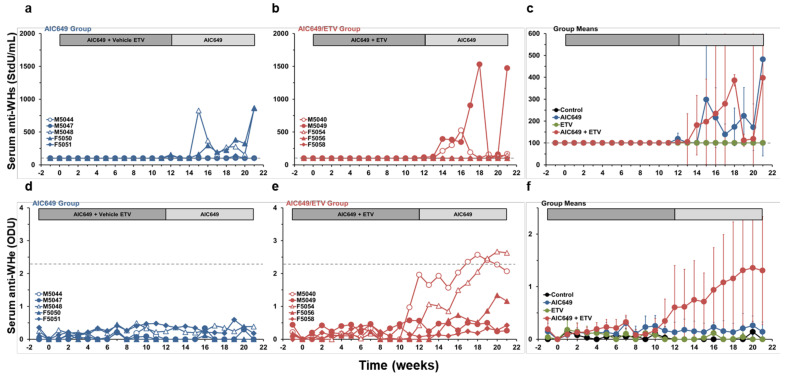
AIC649 monotreatment and AIC649/ETV combination treatment elicits WHV-specific antibodies in a subset of woodchucks. Changes in serum anti-WHs antibody titers and anti-WHe antibody levels relative to pretreatment (week −1 and T0) in woodchucks during (**a**,**d**) AIC649 monotreatment, (**b**,**e**) AIC649/ETV combination treatment and (**c**,**f**) means of experimental groups. The horizontal dotted line indicates the detection limit for anti-WHs antibodies (i.e., 100 StdU/mL) in the top panels and for anti-WHe antibodies (i.e., 2.286 ODU) in the bottom panels. The vertical lines represent the standard error of the mean. Anti-WHs titers were comparable between the Control, AIC649, ETV, and AIC649/ETV Groups (*p* > 0.05). Anti-WHe antibody levels were significantly increased compared to the Control Group during weeks 17–21 in the AIC649/ETV Group (*p* < 0.001), but not in the AIC649 and ETV Groups (*p* > 0.05). Anti-WHe antibody levels were comparable between the AIC649, ETV, and AIC649/ETV Groups (*p* > 0.05). Abbreviations: ODU, optical density unit; StdU, standard unit.

Elicitation of anti-WHs antibodies in serum was noted for three woodchucks each administered AIC649, alone and in combination with ETV (Figure 6). Anti-WHs antibodies were mainly detected during maintenance treatment but were elicited as early as during the last week of active treatment and correlated with the prolonged reduction or undetectability of serum WHsAg in most animals. One exception noted was woodchuck M5049 of the AIC649/ETV Group that presented with the highest antibody titer although the reductions in serum WHV DNA, WHsAg, and WHeAg were less pronounced than in other animals of this group. Detection of anti-WHs antibodies was transient in one woodchuck of each the AIC649 and AIC649/ETV Groups and more durable in the other two animals of both groups, with low to moderate titers of around 1000 StdU/mL. Elicitation of anti-WHe antibodies in serum, however, was only observed in two woodchucks that underwent AIC649/ETV combination treatment and that had prior loss of WHeAg. Detection of anti-WHe antibodies was transient in one woodchuck and durable in the second animal. Since anti-WHs and anti-WHe antibodies were not detected during placebo treatment and ETV monotherapy, this indicated that immunomodulation with AIC649, alone and in combination with ETV, induced an adaptive immune response in woodchucks.

### 3.5. AIC649 Treatment, Alone and in Combination with ETV, was Well Tolerated in Woodchucks

**Figure 7:**
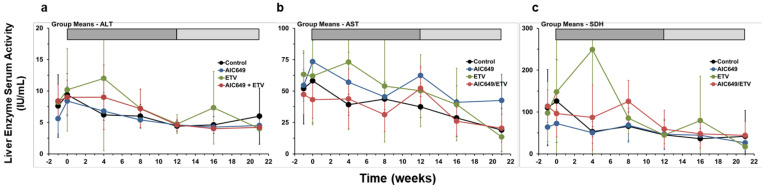
AIC649 monotreatment and AIC649/ETV combination treatment results in minor but transient elevations in the serum activity of liver enzymes. Comparison of means of the Control, AIC649, ETV, and AIC649/ETV Groups relative to pretreatment (week −1 and T0) for serum levels of (**a**) alanine aminotransferase (ALT), (**b**) aspartate aminotransferase (AST), and (**c**) sorbitol dehydrogenase (SDH). The vertical lines represent the standard error of the mean. ALT levels were comparable between the Control, AIC649, ETV, and AIC649/ETV Groups (*p* > 0.05). AST levels were significantly increased compared to the Control Group at week 12 in the AIC649 Group (*p* < 0.001) but not in the ETV and AIC649/ETV Groups (*p* > 0.05). The AST level was significantly reduced compared to the AIC649 Group at T0 in the AIC649/ETV Group (*p* < 0.05). AST levels were comparable between the ETV and AIC649/ETV Groups (*p* > 0.05). SDH levels were comparable between the Control, AIC649, ETV, and AIC649/ETV Groups (*p* > 0.05). Abbreviation: IU, international units.

Pronounced elevations in the serum activity of liver enzymes occurred only during ETV monotreatment in one woodchuck of the ETV Group and the increases in ALT, AST, and SDH levels apparently coincided with the initial period of viral load reduction, indicating most likely a nucleoside-associated effect (Figure 7 and Figures S7–S9). Two other woodchucks in this group and one animal of the Control Group also had more marked increases in the levels of all three liver enzymes that were noted shortly before the scheduled euthanasia due to the development of large liver tumors/end-stage HCC, indicating most likely a hepatocellular effect. Other but minor increases in liver enzymes were occasionally observed during AIC649 monotherapy and AIC649/ETV combination treatment, suggesting an immunomodulator-associated effect. Elevations in liver enzyme levels were present in one to two animals each in the AIC649 and AIC649/ETV Groups during (ALT and SDH) or at the end of active treatment (AST). Of note is that these increases did not always occur in the same woodchuck; however, during AIC649/ETV combination treatment they were only present in animals with suppressed or undetectable WHV markers in the periphery and liver. Nevertheless, the liver enzyme elevations during active treatment were not significantly different between the AIC649, ETV, and AIC649/ETV Groups, reversed thereafter and normalized during maintenance treatment.

AIC649 treatment of woodchucks, alone and in combination with ETV, was safe as no mortality occurred during active and maintenance treatment with the immunomodulator. Since both treatment regimens did not adversely affect body weights, body temperatures, and hematology and clinical chemistry parameters (data not shown), this overall supported the safety of immunomodulation with AIC649 in woodchucks with CHB.

### 3.6. AIC649 Treatment, Alone and in Combination with ETV, Induced Tightly Regulated Immunological and Virological Responses During Active and Maintenance Treatment

**Figure 8:**
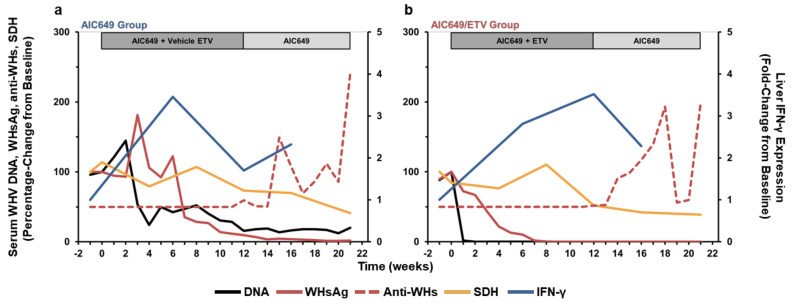
AIC649 monotreatment and AIC649/ETV combination treatment induce tightly regulated immunological and virological responses during active and maintenance treatment. Comparison of group means relative to pretreatment (week −1 or T0) during (**a**) AIC649 monotreatment and (**b**) AIC649/ETV combination treatment for serum WHV DNA, WHsAg, anti-WHs antibodies, and SDH and intrahepatic IFN-γ expression. The group mean for parameters on the left *y*-axis is presented as a percentage-change, while the group mean for parameters on the right *y*-axis is presented as a fold-change.

The comparison of antiviral effects on serum viremia and antigenemia with intrahepatic innate and peripheral adaptive immune responses revealed a tightly regulated response pattern to treatment with the immunomodulator AIC649, alone and in combination with ETV (Figure 8). During AIC649 monotreatment, the minor increases in serum WHV DNA and WHsAg shortly after the initiation of active treatment correlated with an increased expression of type I and II IFNs, but was without parallel elevation in liver enzymes, such as SDH. As IFN expression was tested for the first time at week 6, it is unknown if the transient increases in viremia and antigenemia during the initial weeks of AIC649 treatment were preceding or coinciding with the innate immune response. During AIC649/ETV combination treatment, these elevations in viremia and antigenemia were not present, most likely due to the fast-acting, antiviral activity of the nucleoside analog. Correlating with the peak in IFN expression at week 6 and the subsequent decline at week 12, WHV DNA and WHsAg loads declined in the AIC649 Group but the reductions were only modest in most animals. In the AIC649/ETV Group, the reductions in viremia and antigenemia were more pronounced and correlated with a sometimes higher and but always longer-lasting IFN expression that peaked at week 12. Since minor elevations in SDH level were also present in both groups at week 8, this overall could suggest liver inflammation during active treatment due to immune-mediated control of the replicating virus in hepatocytes by non-cytolytic and cytolytic mechanisms. After the switch to maintenance treatment, IFN expression in the AIC649 Group increased again slightly, while viremia and antigenemia continued to stay suppressed or started to decline more markedly in some animals and anti-WHs antibodies were detected for the first time. In the AIC649/ETV Group, IFN expression declined during maintenance treatment but was still elevated, when compared to the pretreatment baseline. Viremia and especially antigenemia became maximal suppressed or even undetectable in most animals during this time and anti-WHs antibodies were elicited. Since no additional increase in SDH level was noted in both groups, this could suggest continued immune-mediated viral control during maintenance treatment by a non-cytolytic mechanism, in addition to the developing humoral response.

## 4. Discussion

The present study evaluated the antiviral efficacy and safety of AIC649 in woodchucks during 21 weeks of treatment using initially iv administration for active treatment and then im administration for maintenance treatment. Since most immunotherapeutic approaches for CHB will be tested on top of conventional antiviral treatment with NAs, AIC649 was further evaluated in combination with the nucleoside analog ETV that was administered po during active treatment.

In comparison to placebo treatment, AIC649 monotherapy produced modest but significant reductions in viremia in all woodchucks that were in the same range as previously observed in this animal model [63]. Different to the previous study, however, was that pronounced declines in surface and e antigenemia occurred in a few animals, especially during maintenance treatment. These changes were associated with an elicitation of antibodies against WHsAg only, but were without significant reductions in intrahepatic WHV replication. The greater antiviral effect of AIC649 on antigenemia was likely due to the initial iv administration and longer treatment duration, resulting in a larger and prolonged stimulation of DCs in liver and release of Th1 cytokines, such as type I and II IFNs, as also described for human blood cells and mice treated with this immunomodulator [56,58]. The antiviral effect in woodchucks further appeared to depend on the development of an antibody response by B-cells, possibly primed by stimulated DCs and/or triggered directly by AIC649 via TLR9 stimulation to secrete immunoglobulins [14]. However, there was variation in the individual response that may relate to the outbred nature of woodchucks and the severity of impaired DC functions caused by CHB in these animals, as also observed in other studies that evaluated TLR7/8 agonists in this animal model [48,70]. A limited antiviral activity of an immunomodulator stimulating TLR9, when used as a single agent, has also been observed in a separate woodchuck study [71]. Weekly subcutaneous administration of a CpG oligonucleotide for 16 weeks resulted in minor reductions in WHV DNA but had no effect on WHsAg, and viral relapse occurred during treatment. The greater and more durable antiviral effect mediated by AIC649 than by CpG is likely due to the additional triggering of TLR-independent pathways by this immunomodulator [54,55,57,58,59], leading to the stimulation of cDCs, in addition to pDCs, and to a greater priming/activation of antiviral NK-, B-, and T-cells [56,58]. The effect on viremia achieved by AIC649 and CpG monotreatment in woodchucks, however, was lower than comparable treatment regimens in HBV transgenic mice [58,62,63,72,73], suggesting a greater responsiveness of mice than woodchucks to at least TLR9 stimulation.

ETV monotreatment produced fast and marked reductions in viremia in all woodchucks but did not significantly affect surface and e antigenemia. The antiviral effect was transient and WHV DNA relapsed immediately after drug withdrawal. Strong antiviral efficacy by ETV is typically achieved in woodchucks and recrudescence of viral replication after treatment discontinuation is a common observation [70,71,74,75], as also reported for patients with CHB [76], indicating that viremia suppression is dependent on the continued presence of this nucleoside analog. ETV monotreatment in woodchucks at low dosage, as applied in the present study, usually does not reduce circulating WHsAg and intrahepatic WHV cccDNA and RNA nor elicits WHV-specific antibodies [44,71]. This is comparable to the situation in most patients undergoing standard antiviral treatment with NAs, including ETV [5,6]. 

Compared to both monotreatment regimens, AIC649/ETV combination treatment produced a long-lasting suppression or even undetectability of viremia and surface and e antigenemia during active and maintenance treatment that correlated with significant reductions in intrahepatic WHV replication in most woodchucks and with the elicitation of antibodies to WHsAg and WHeAg in a subset of animals. The greater antiviral effect is likely due to a functional recovery of APC subsets by ETV, as reductions in viremia by the nucleoside analog Adefovir resulted in the improvement of cDC (but not of pDC) functions in patients with CHB, including increases in cell frequency, IL-12 production, and T-cell stimulatory capacity [64]. Parallel AIC649 treatment may have stimulated these functionally recovered cDCs, leading to an increased priming/activation of antiviral immune cells. The latter cells, in addition to the released Th1 cytokines, then likely contributed to a pronounced reduction in WHV replication in liver, which in turn may have allowed the functional recovery of pDCs after removal of the tolerizing viral surface and e proteins and their stimulation by AIC649. Following ETV withdrawal, AIC649 stimulation of both APC subsets continued, and thus was likely able to preserve or to enhance immunological control of the viral infection in absence of the nucleoside analog. However, the antiviral effect mediated by combination treatment was not uniform in all woodchucks. Animal M5049 also experienced a marked decline in WHV DNA and to a lesser degree in WHsAg and WHeAg, but the effect on viremia was transient and the immediate relapse after ETV withdrawal was comparable to animals that underwent ETV monotherapy. This suggests that this particular woodchuck responded differently to AIC649 treatment for unknown reasons, including an anti-WHs antibody titer that was the highest among all animals that developed an antibody response. A superior antiviral activity of a TLR9-stimulating immunomodulator in combination with ETV was also recognized during CpG treatment of woodchucks [71]. CpG administration for 16 weeks together with daily po ETV dosing for 12 weeks resulted in undetectable viremia in all animals and WHV DNA suppression was more durable than during ETV monotherapy. Different to AIC649/ETV combination treatment, CpG/ETV-mediated suppression occurred earlier than during ETV monotherapy, suggesting a benefit of targeted TLR9 agonism over a broader stimulation of TLR9-dependent and TLR-independent pathways in the rapid reduction of viral DNA. CpG/ETV combination treatment also mediated WHsAg loss in most animals, but different to AIC649/ETV, was not associated with seroconversion to anti-WHs antibodies, suggesting a benefit of AIC649 in priming and/or stimulation of B-cells via TLR9. A greater antiviral efficacy of combination treatment over ETV monotherapy was further demonstrated in woodchucks for various immunomodulators and checkpoint inhibitors [44,70,75,77,78,79].

Induction of an innate immune response in woodchucks by AIC649 was tested via the expression of type I and II INFs in liver. Placebo treatment was not associated with changes in the intrahepatic transcription of IFN-α, IFN-β, and IFN-γ. This is consistent with a diminished or impaired antiviral immune response usually present during CHB in woodchucks [28,33,35,80,81,82] and patients [18,83,84], suggesting that the high levels of virions and subviral, surface-containing particles, and possibly of e antigen, are implicated in the maintenance of immunologic tolerance against hepadnaviruses at multiple levels, including DCs [50,51,52]. In agreement with this, ETV had no effect on antigenemia in the present study, and thus was unable to broadly induce IFNs in woodchucks. The occasional but minor elevations in IFN transcription at the end of drug treatment could indicate some unmasking of the underlying but deficient antiviral immunity, possibly by functionally recovered cDCs, in response to the marked suppression of viremia, as also observed for ETV in another woodchuck study [77]. Since IFN expression was also somewhat elevated in a few animals after ETV withdrawal, this could indicate an additional antiviral immune mechanism triggered by recrudescence of viral replication, as reported recently in woodchucks [70]. One limitation of the current study is that changes in intrahepatic IFN expression during and after ETV treatment could only be fully studied in two woodchucks, as the other three animals of this group were euthanized at various time points due to severe HCC development. Thus, the possible contribution of the nucleoside analog to the induction and magnitude of IFN transcription during AIC649/ETV combination treatment could not firmly be established.

However, the induction of IFN expression by AIC649 during mono and combination treatment was temporally different to ETV. Consistent with the stimulation of TLR9-dependent and TLR-independent pathways in DCs by the immunomodulator [54,55,56,58], IFN-α and IFN-β transcription increased transiently in most animals during active treatment. The same kinetic was observed for IFN-γ that was most likely expressed in liver resident or infiltrating immune cells, such as NK- and/or T-cells [56], in response to the induced type I IFNs. The subsequent decline in type I and II IFN expression during monotreatment could indicate that continued stimulation of DCs by AIC649 in the setting of high WHV replication was somehow refractory. This could further explain why animals with more pronounced changes in surface and e antigenemia during maintenance treatment had a second increase in IFN transcription during this time, most likely by functionally recovered pDCs. The higher magnitude of IFN expression during active treatment with AIC649 in combination with ETV and its longer presence during maintenance treatment correlated well with the marked effects on surface and e antigenemia in most animals, indicating that the setting of low WHV replication mediated by ETV was beneficial for the continued stimulation of p and cDCs by AIC649. Induction of an antiviral innate immune response was also obtained during CpG treatment of HCV-infected patients and woodchucks with CHB based on increased IFN levels in serum and elevated presence of IFN-stimulated genes in blood [71,85]. These changes were sometimes rather durable in individual woodchucks but were not different between the groups that received CpG monotherapy or CpG/ETV combination treatment, while they were essentially absent in the group treated with ETV alone. Blood IFN-β expression appeared more mixed, but comparable to the present study, it was markedly increased in individual animals, and more so during combination treatment than during monotherapy.

Of interest was that the current study confirmed the previously observed biphasic pattern of antiviral response to AIC649 immunomodulation [63], despite using a different administration route during active treatment. The initial increase in WHV DNA and WHsAg in the previous study was accompanied by elevations in peripheral WHV-specific T-cells and Th1 cytokines. This rise in serum viremia and antigenemia, including WHeAg, was also noted in the current study, in addition to the increase in intrahepatic IFN expression. Thereafter, viral markers declined transiently in both studies and then continued to decline further albeit at varying degrees. The AIC649-induced biphasic response pattern in woodchucks appears comparable to the increases in HBV DNA and/or elevations in liver enzymes that sometimes precede the induction of an antiviral immune response in patients spontaneously resolving HBV infection [86,87]. This biphasic pattern could have translational relevance in predicting antiviral response to AIC649 treatment in patients in case future clinical administration is performed as monotherapy or as add-on treatment (i.e., before or after NA therapy). However, if AIC649 is administered on top of conventional NA treatment as assessed in the present woodchuck study, this biphasic response pattern will most likely not be present.

Together with the minor but transient elevations in serum liver enzymes that were only observed during AIC649 monotherapy and AIC649/ETV combination treatment, and especially in animals with suppressed or undetectable viremia and/or antigenemia thereafter, this suggested that an antiviral immune response critical for attaining initial control over the virus was induced shortly after treatment initiation. This immune-mediated viral control appeared to consist of non-cytolytic and cytolytic mechanisms. Although not determined directly, but described for TLR9 stimulation in general [12,14] and for AIC649 immunomodulation in particular [54,56,58], these mechanisms may involve type I IFNs that are produced by pDCs and cDCs and type II IFNs that are expressed by NK- and/or T-cells. Importantly, IFN-α can inhibit the transcription of pgRNA from cccDNA, block its packaging into nucleocapsids and prevent viral replication through upregulation of a ribonuclease, thereby contributing to the overall suppression of chronic HBV infection [88,89,90]. These mechanisms may further involve the cytotoxic activity of immune cells, such as NK-cells activated by IFN-dependent and -independent pathways and CTLs induced by DCs leading to the elimination of virus-infected hepatocytes. Following the switch to maintenance treatment, IFN expression in liver increased again during AIC649 monotherapy in a few woodchucks or stayed elevated during AIC649/ETV combination treatment in most animals with pronounced antiviral effect, but the innate immune response was not associated with additional elevations in liver enzymes. This suggested continued immune-mediated viral control by a non-cytolytic mechanism, in addition to the elicitation of antibodies against WHsAg and/or WHeAg by plasma B-cells. Seroconversion to WHV-specific antibodies is typically a rare event in woodchucks with CHB, but was also achieved during treatment with other immunomodulators, administered alone or in combination with ETV [44,48,70,79,80,91].

As mentioned above, AIC649 monotreatment was associated with the induction of a T-cell response to the viral core and surface antigens in woodchucks of the previous study [63]. Thus, a second limitation of the current study is that AIC649-mediated WHV-specific T-cell responses are not presented. However, together with the antibody response by surface and e antigen specific plasma B-cells observed in the current study, this suggested a crosslink between innate and adaptive immunity. Development of virus-specific cellular and humoral responses may indicate an improvement in the underlying and/or a retrieval of the antiviral immunity in woodchucks with CHB. As established in the present study, stimulation of TLR9-dependent and TLR-independent pathways in woodchucks by AIC649 treatment induced type I and II IFN expression in the liver and elicited WHV-specific antibodies in the periphery. The underlying mechanism is most likely a reversal of the diminished or impaired functions of DCs in these animals that resulted in the activation of virus-specific B- and T-cells, as it was also concluded from woodchuck studies that evaluated other immunomodulators [48,70,80,91]. Similar to CpG-mediated TLR9 stimulation of pDCs [14] and comparable to AIC649-mediated TLR-independent stimulation of cDCs [54,55], immunomodulation in woodchucks led to the maturation and differentiation of these APC subsets, resulting in an increased production and secretion of type I IFNs. These IFNs then led to improved antigen processing and presentation by DCs via upregulation of MHC I and II molecules and costimulatory receptors, as shown for AIC649-treated murine DCs [54], that are needed for cross-priming and activation of Th1 cells and CTLs. In addition, TLR9 is also present in B-lymphocytes and activation of the B-cell antigen receptor together with TLR9 by AIC649 possibly induced their differentiation into immunoglobulin-secreting plasma cells, as it has been described for CpG-containing DNA [92]. Since the antiviral efficacy of AIC649/ETV combination treatment was superior over AIC649 and ETV monotherapy, this indicated that an initial or parallel reduction of viremia by the nucleoside analog was likely a requirement for the activity of the immunomodulator, involving functional recovery and stimulation of cDCs initially and of pCDs subsequently, as described above. This further indicated that a combination of therapeutic interventions targeting different steps in the life cycle of WHV, and by analogy of HBV, may be able to address the known limitations of conventional antiviral treatment with NAs.

All applied treatment regimens involving AIC649 were not associated with treatment-limiting adverse effects and extended iv or im administration of the immunomodulator was well-tolerated by woodchucks. The induced immune-mediated viral control was not accompanied by severe liver injury, such as necrosis. Furthermore, signs of systemic immunotoxicity related to changes in hematology, such as thrombocytopenia, neutropenia, or anemia, and clinical chemistry, such as sustained elevations in the levels of bilirubin and liver enzymes, were absent. Regarding the latter, the safety profile of AIC649 appears distinct to CpG oligonucleotides [71], as hepatic flares based on marked and durable elevations in liver enzymes were not noted. Since the AIC649-induced Th1 and inflammatory responses are limited by a subsequent upregulation of Th2 and anti-inflammatory responses [56], this may have attenuated immunopathogenesis in woodchucks, as also observed in other animal models during treatment with the immunomodulator [58]. Absence of liver damage in woodchuck may further relate to an AIC649-induced downregulation of viral antigen cross-presentation of liver sinus endothelial cells to activated virus-specific CTLs, as observed during immunomodulation in HBV transgenic mouse models [62].

The safety, tolerability, and pharmacodynamics of AIC649 were already evaluated in patients with CHB during an ascending dose phase I clinical trial [93]. A single iv dose of the immunomodulator was well-tolerated and dose-limiting toxicity was not observed, including the highest dose administered to patients that equaled to the dose administered repeatedly to woodchucks of the current study. Furthermore, a single AIC649 dose induced an innate immune response in the blood of patients, as determined by the increases in immune-regulating cytokines, including various interleukins and IFN-γ. Thus, the frequency and duration of AIC649 administration established in the present woodchuck study for safety and antiviral efficacy needs to be confirmed in patients, and eventually modified, as one animal that underwent combination treatment with ETV did not achieve a complete response. Nonetheless, the results obtained in the woodchuck animal model of CHB suggest that the immunomodulator AIC649 in combination with the conventional nucleoside analog ETV has the potential to safely induce immune-mediated HBV control in patients and support the continued development of this combination regimen for treatment and possibly functional cure of chronic HBV infection.

## Data Availability

All relevant data generated during the study are presented within the manuscript and the Supplementary Materials.

## Supplementary Materials

The following are available online at https://www.mdpi.com/article/10.3390/v13040648/s1, Table S1: Primer and probe sets used for detection of IFNs in woodchuck liver, Figure S1: AIC649/ETV combination treatment results in suppression or undetectability of serum e antigenemia in most woodchucks, Figure S2: AIC649/ETV combination treatment results in suppression or undetectability of WHV DNA RI in the liver, Figure S3: AIC649/ETV combination treatment results in suppression or undetectability of WHV RNA in the liver, Figure S4: AIC649/ETV combination treatment results in enhanced expression of IFN-α in the liver, Figure S5: AIC649/ETV combination treatment results in enhanced expression of IFN-β in the liver, Figure S6: AIC649/ETV combination treatment results in enhanced expression of IFN-γ in the liver, Figure S7: AIC649 monotreatment and AIC649/ETV combination treatment results in minor but transient elevations in the serum activity of ALT, Figure S8: AIC649 monotreatment and AIC649/ETV combination treatment results in minor but transient elevations in the serum activity of AST, Figure S9: AIC649 monotreatment and AIC649/ETV combination treatment results in minor but transient elevations in the serum activity of SDH, Methods.
