# Supplementary material for: Treatment with the Immunomodulator AIC649 in Combination with Entecavir Produces Antiviral Efficacy in the Woodchuck Model of Chronic Hepatitis B
Source: Viruses. 2021 Apr 9;13(4):648. doi: 10.3390/v13040648 (PMC8069054; doi:10.3390/v13040648)
Supplement: Supplementary file 1 [file viruses-13-00648-s001.pdf]

Supplementary Data

1. Tables and Figures

Table 1. Primer and probe sets used for detection of IFNs in woodchuck liver.

Gene	Primers and Probe	Sequence
	F	5′-CTCAAGCTGTTGCTGTCCTC-3′
IFN-α	R	5′-CTTCTGGGTGCTGAAGAGGT-3′
	P	5′-CCAGATGACCCAGCAGATCCTCA-3′
	F	5′-GAATGAAAGGCCTGCAGAGT-3′
IFN-β	R	5′-GAATGTTTGATCTCCTTGGG-3′
	P	5′-CTTGAAGTCCATCCTGTCACTGAGGC-3′
	F	5′-ATCCAAAGGAGCATGGACAC-3′
IFN-γ	R	5′-TGAACTTGAGACACCTTTAGGAA-3′
	P	5′-CAACAGCAGTACCAATAAGCTGCAGGA-3′
	F	5′-GTAACCCGTTGAACCCCATT-3′
18S rRNA	R	5′-GGGACTTAATCAACGCAAGC-3′
	P	5′-GCAATTATTCCCCATGAACG-3′

F: forward primer, R: reverse primer, P: probe.

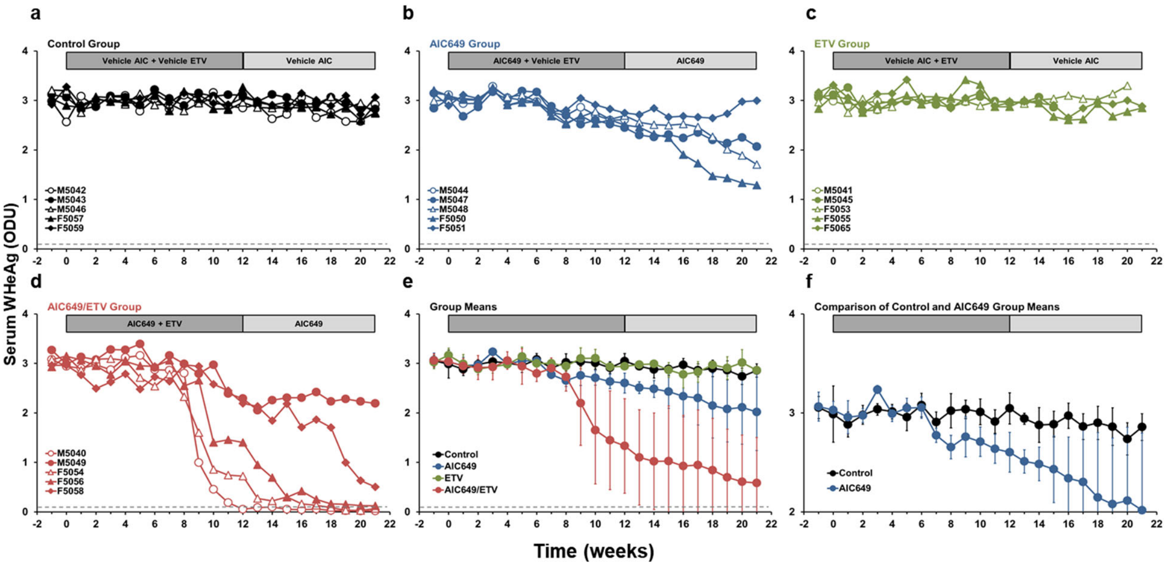

Figure 1. AIC649/ETV combination treatment results in suppression or undetectability of serum e antigenemia in most woodchucks. Changes in serum WHeAg levels relative to pretreatment (week −1 and T0) in woodchucks during (**a**) placebo treatment, (**b**) AIC649 monotreatment, (**c**) ETV monotreatment, (**d**) AIC649/ETV combination treatment, and (**e**) group means. (**f**) The comparison of means of Control and AIC649 Groups illustrates the biphasic change in serum WHeAg level during initial AIC649 monotreatment. The vertical lines represent the standard error of the mean. The horizontal dotted line indicates the detection limit for WHeAg (i.e., 0.097 ODU). The vertical lines represent the standard error of the mean. Antigenemia levels were significantly reduced compared to the Control Group during weeks 7–13 and at week 16 in the AIC649 Group ($p < 0.05$) and at week 6 and during weeks 10–21 in the AIC649/ETV Group ($p < 0.05$) but not in the ETV Group ($p > 0.05$). In addition, the antigenemia level was significantly increased compared to the Control Group at week 3 in the AIC649 Group ($p < 0.001$). Antigenemia levels were also significantly reduced compared to the AIC649 Group at week 3 in the ETV Group ($p < 0.05$) and at weeks 3 and 6 and during weeks 11–21 in the AIC649/ETV Group ($p < 0.05$). The antigenemia level was further significantly reduced compared to the ETV Group during weeks 10–21 in the AIC649/ETV Group ($p < 0.05$).

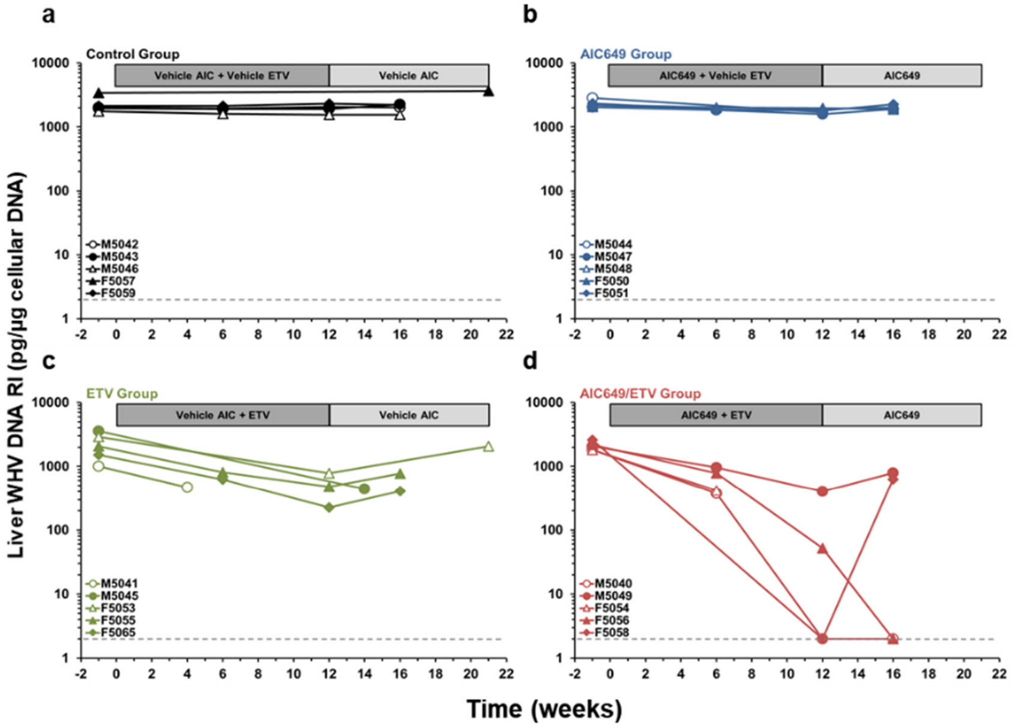

Figure 2. AIC649/ETV combination treatment results in suppression or undetectability of WHV DNA RI in the liver. Changes in intrahepatic WHV DNA RI levels relative to pretreatment (week −1) in woodchucks during (**a**) placebo treatment, (**b**) AIC649 monotreatment, (**c**) ETV monotreatment, and (**d**) AIC649/ETV combination treatment. The horizontal dotted line indicates the detection limit for WHV DNA RI (i.e., 2.0 pg/µg cellular DNA).

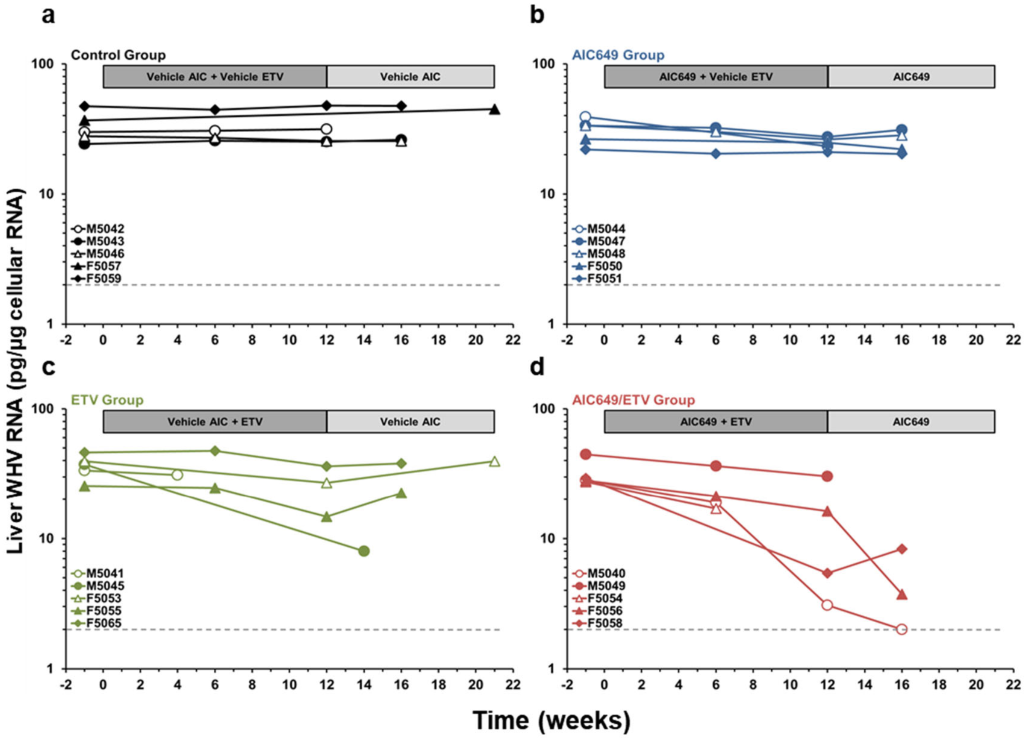

Figure 3. AIC649/ETV combination treatment results in suppression or undetectability of WHV RNA in the liver. Changes in intrahepatic WHV RNA levels relative to pretreatment (week −1) in

woodchucks during (**a**) placebo treatment, (**b**) AIC649 monotreatment, (**c**) ETV monotreatment, and (**d**) AIC649/ETV combination treatment. The horizontal dotted line indicates the detection limit for WHV RNA (i.e., 2.0 pg/μg cellular RNA).

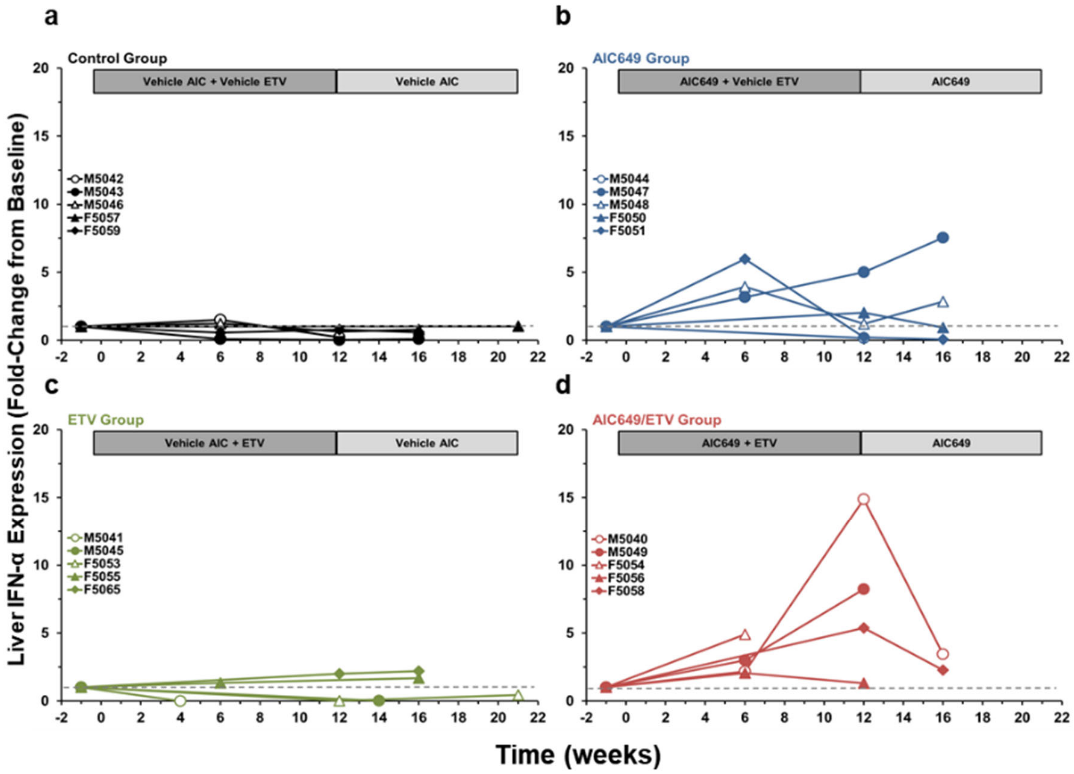

Figure 4. AIC649/ETV combination treatment results in enhanced expression of IFN-α in the liver. Changes in intrahepatic IFN-α transcript levels relative to pretreatment (week −1) in woodchucks during (**a**) placebo treatment, (**b**) AIC649 monotreatment, (**c**) ETV monotreatment, and (**d**) AIC649/ETV combination treatment. Fold-change values were calculated relative to the pretreatment baseline, which was set a 1.0 and is indicated by the horizontal dotted line.

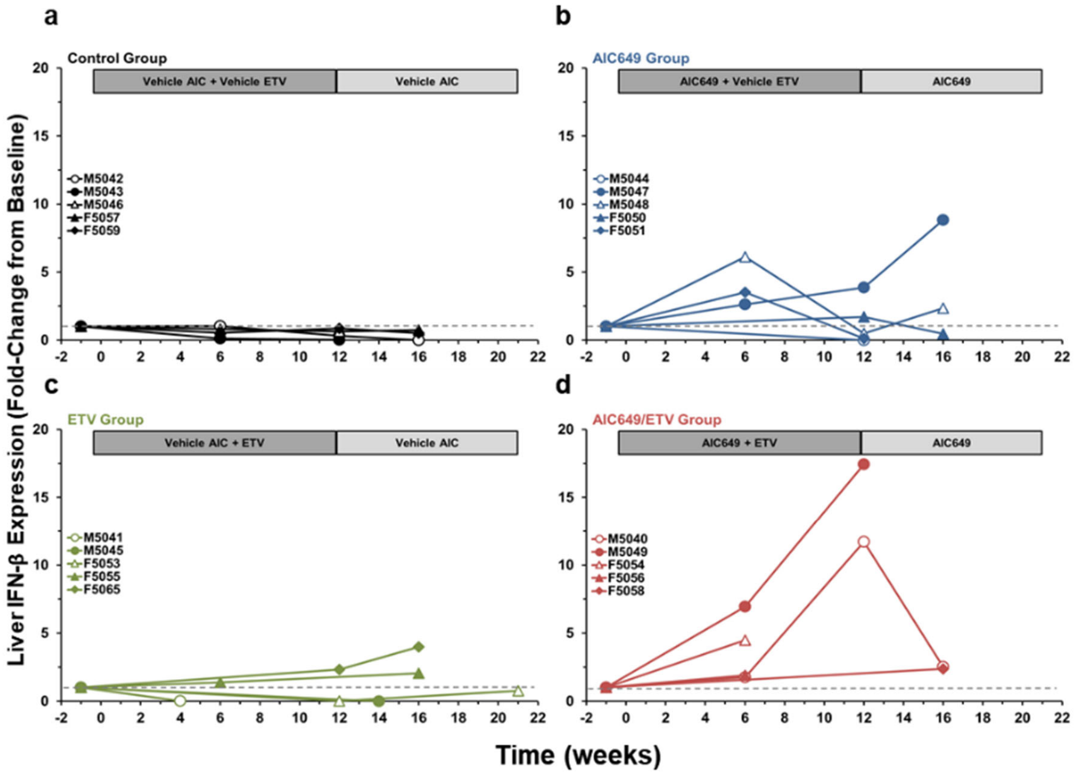

Figure 5. AIC649/ETV combination treatment results in enhanced expression of IFN-β in the liver. Changes in intrahepatic IFN-β transcript levels relative to pretreatment (week −1) in woodchucks during (**a**) placebo treatment, (**b**) AIC649 monotreatment, (**c**) ETV monotreatment, and (**d**) AIC649/ETV combination treatment. Fold-change values were calculated relative to the pretreatment baseline, which was set a 1.0 and is indicated by the horizontal dotted line.

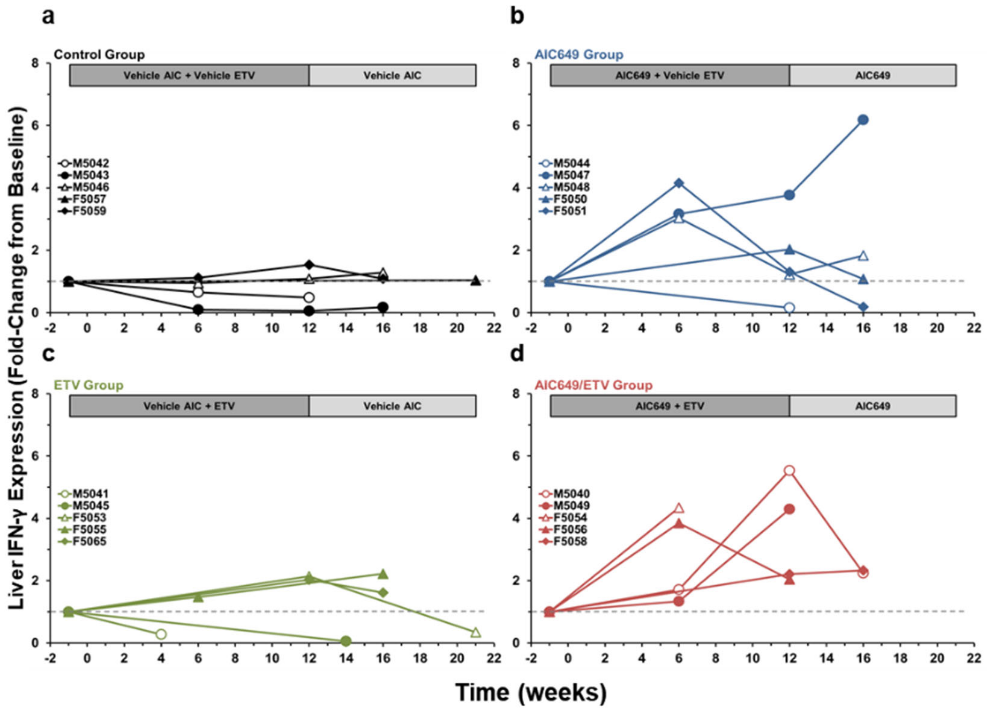

Figure 6. AIC649/ETV combination treatment results in enhanced expression of IFN-γ in the liver. Changes in intrahepatic IFN-γ transcript levels relative to pretreatment (week −1) in woodchucks

during (**a**) placebo treatment, (**b**) AIC649 monotreatment, (**c**) ETV monotreatment, and (**d**) AIC649/ETV combination treatment. Fold-change values were calculated relative to the pretreatment baseline, which was set a 1.0 and is indicated by the horizontal dotted line.

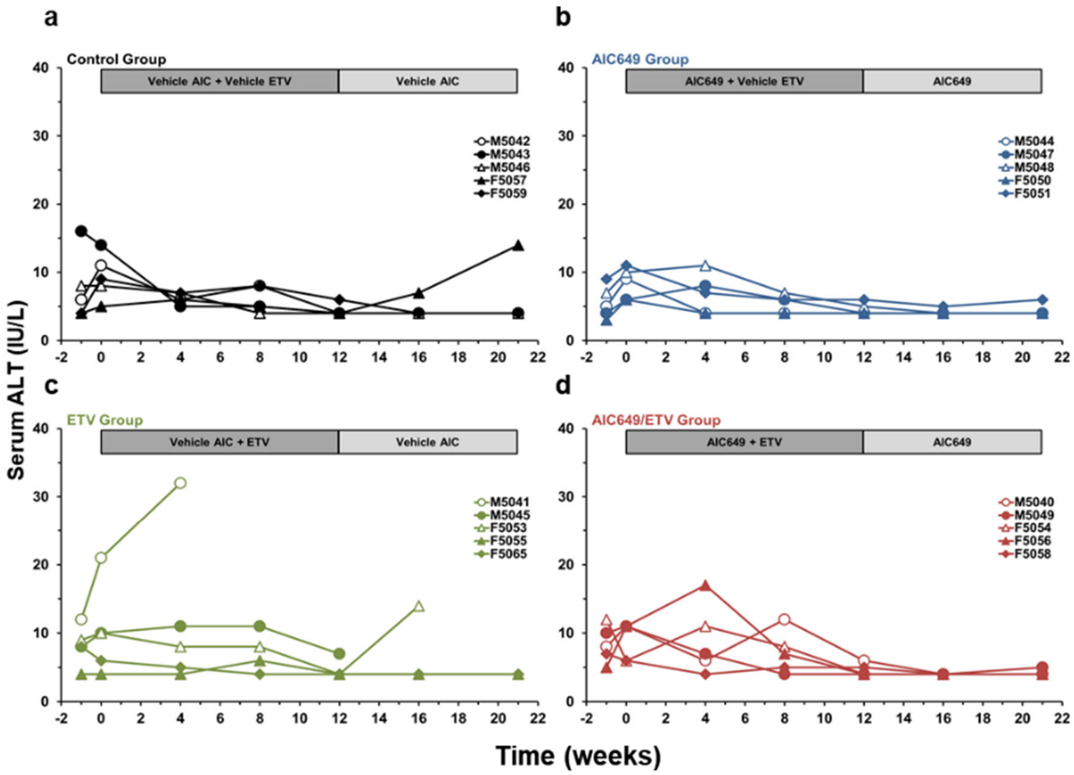

Figure 7. AIC649 monotreatment and AIC649/ETV combination treatment results in minor but transient elevations in the serum activity of ALT. Changes in serum ALT levels relative to pretreatment (week −1 and T0) in woodchucks during (**a**) placebo treatment, (**b**) AIC649 monotreatment, (**c**) ETV monotreatment, and (**d**) AIC649/ETV combination treatment. Abbreviation: IU, international units.

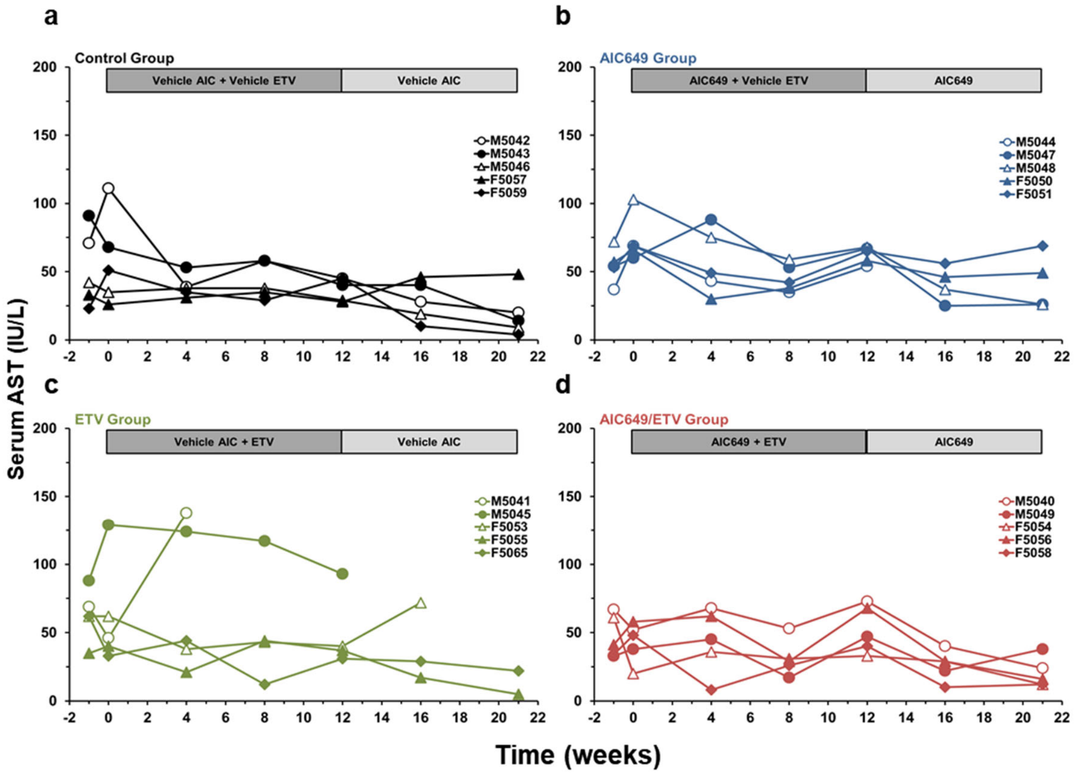

Figure 8. AIC649 monotreatment and AIC649/ETV combination treatment results in minor but transient elevations in the serum activity of AST. Changes in serum AST levels relative to pretreatment (week −1 and T0) in woodchucks during (**a**) placebo treatment, (**b**) AIC649 monotreatment, (**c**) ETV monotreatment, and (**d**) AIC649/ETV combination treatment. Abbreviation: IU, international units.

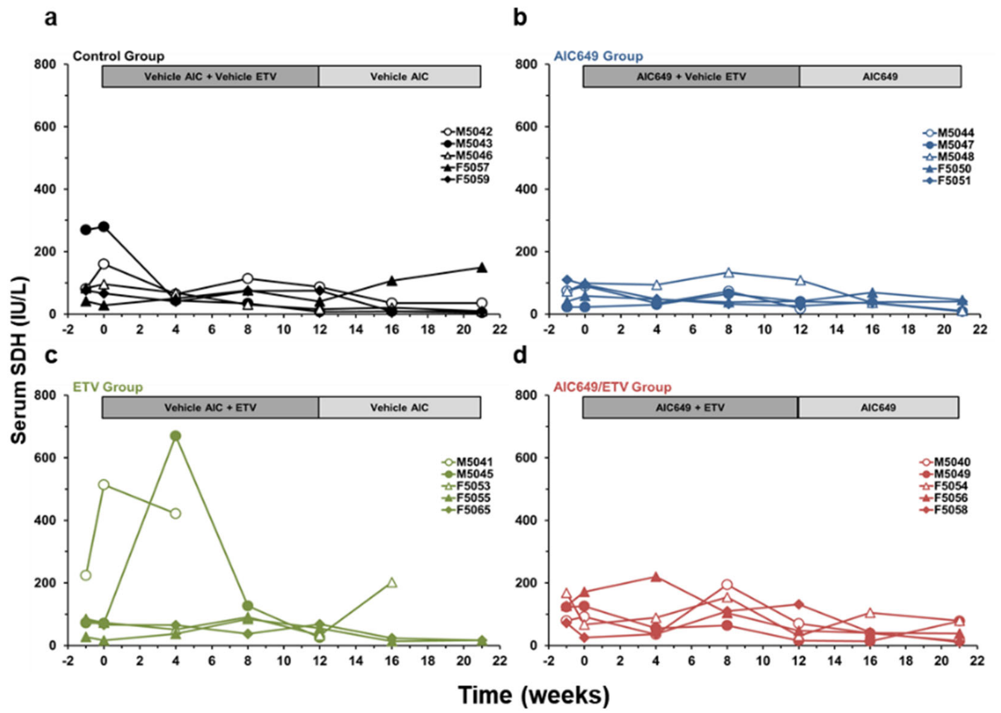

Figure 9. AIC649 monotreatment and AIC649/ETV combination treatment results in minor but transient elevations in the serum activity of SDH. Changes in serum SDH levels relative to

pretreatment (week −1 and T0) in woodchucks during (**a**) placebo treatment, (**b**) AIC649 monotreatment, (**c**) ETV monotreatment, and (**d**) AIC649/ETV combination treatment. Abbreviation: IU, international units.

2. Methods

2.1.Serum WHV Parameters

2.1.1. Serum WHV DNA

Triplicate of 10 μL aliquots of woodchuck serum were assayed quantitatively against a 10-fold dilution series of a known standard WHV genome plasmid (pUC-CMVWHV) [1] using slot blot hybridization (lower assay sensitivity, 1×10^7 WHV ge/mL serum). DNA samples were denatured (0.25 M NaOH, 0.5 M NaCl) for 10 min, after which the reaction was stopped (0.25 M NaOH, 0.2 × saline-sodium citrate [SSC]). DNA samplese were then spotted onto nitrocellulose membranes under vacuum pressure using a dot blot manifold. The membranes were prehybridized and then hybridized overnight at 60 °C in PerfectHyb Plus Hybridization buffer (Sigma-Aldrich, St. Louis, MO) using a ^{32}P-labeled fragment of purified WHV core DNA derived from pUC-CMVWHV. Membranes were then washed extensively (2× SSC ± 1% sodium dodecyl sulfate [SDS]), airdried, and analyzed by radio imaging.

If samples from woodchucks registered below the LLOD of the slot blot assay, 200 μL of serum was then extracted for DNA with the QIAmp DNA Mini Kit (Qiagen) by following the manufacturer's protocol and assayed quantitatively in triplicate on a QuantStudio 3 Real Time PCR System instrument against a 10-fold dilution series of pUC-CMVWHV (lower assay sensitivity, 6.0×10^2 WHV ge/mL serum). TaqMan Gene Expression Master mix (Applied Biosystems) was used by following the manufacturer's protocol and the following primer/probe sets (Integrated DNA Technologies, Coralville, IA):

F: 5′-AGAAGACGCACTCCCTCTCCT-3′,

R: 5′-TGGCAGATGGAGATTGAGAGC-3′, and

P: 5′-AGAAGATCTCAATCACCGCGTCGCAG-3′.

The concentration of each primer was 900 nM, while the concentration of the TaqMan probe was 250 nM. The PCR reaction conditions were 10 min at 95 °C, followed by 40 cycles of 15 s at 95 °C, and 60 s at 60 °C.

2.1.2. Serum WHsAg

Woodchuck serum was diluted (2% bovine serum albumin (BSA), 0.2% Tween 20, 1% phosphate-buffered saline [PBS]) and assayed quantitatively in triplicate against a 10-fold dilution series of a WHV-infected serum with known concentration of WHsAg using a monoclonal antibody-based ELISA (lower assay sensitivity, 5 ng WHsAg/mL serum). For quantifications, a serum sample required assay at several dilutions (i.e., 1:100, 1:1000, 1:5000, and 1:10,000). Additional assays with lower dilutions (i.e., 1:1, 1:10, 1:25, and 1:50) were performed as needed to confirm and recheck specific serum samples with low OD signal output at the 1:100 dilution. Serum samples that were positive at the more limiting dilution(s) (i.e., having non-saturating OD signals and were >3.1 × background of a WHV-uninfected serum) were compared relative to the standard curve, and then back-calculated to undiluted serum.

2.1.3. Serum Anti-WHs

Woodchuck serum was diluted (2% BSA, 0.2% Tween 20, 1% PBS) and assayed quantitatively in triplicate against a 10-fold dilution series of a WHV-infected serum with known concentration of anti-WHs using an EIA (lower assay sensitivity, 100 StdU of anti-WHs/mL serum). WHsAg purified from a WHV-infected serum [2] was used as the capture antigen. This was followed by readout detection with biotinylated protein G conjugate (Thermo Fisher Scientific, Waltham, MA), streptavidin horseradish peroxidase conjugate (Thermo Fisher Scientific), and o-phenylenediamine dihydrochloride (OPD; Thermo Fisher Scientific) colorimetric reaction. For quantifications, a serum sample required assay at two dilutions (i.e., 1:25 and 1:100). Additional assays with lower dilutions (i.e., 1:1, 1:5, and 1:10) were performed as needed to confirm and recheck specific serum samples with low OD signal output at the 1:25 dilution. Positive serum samples with non-saturating OD signals and >3.1 × background of a WHV-uninfected serum were compared relative to the standard curve, and then back-calculated to undiluted serum. Relative to the standard serum, values between 100 and 200 StdU/mL were considered as trace, 200–300 StdU/mL were very low, 300–500 StdU/mL were low, 500–2000 StdU/mL were moderate, and greater than 2000 StdU/mL were high.

2.2. Liver WHV Parameters

2.2.1. Intrahepatic WHV DNA RI and WHV cccDNA

For isolating total DNA, frozen liver tissues were homogenized in Hirt 1 solution (10 mM Tris, pH 8.3; 10 mM EDTA, pH 8.0) and RNase A (Invitrogen) using a plastic pestle. Following homogenization, Hirt 2 solution (4% SDS) and Pronase (Invitrogen) were added and samples incubated overnight at 60 °C. Total DNA was extracted first with Tris-equilibrated phenol and thereafter with phenol-chloroform/isoamylalcohol using Phase Lock Gel tubes (Eppendorf/Sigma-Aldrich) by following the manufacturer's protocol. The top layer was precipitated with 100% ethanol and 3 M sodium acetate for 30 min at −80 °C. The pellet obtained after centrifugation at 13,000 rpm for 30 min at 4 °C was washed with 70% ethanol and resuspended in nuclease-free water. The total DNA concentration within a sample was determined with the Nano DropTM 8000 spectrophotometer (Thermo Fisher Scientific). Thereafter, 2 µg of total DNA was digested with Hind*III* (300 units in 10× buffer [both from Invitrogen]) and incubated overnight at 37 °C. Digested DNA was precipitated with 100% ethanol and 3 M sodium acetate for 30 min at −80 °C. The pellet obtained after centrifugation at 13,000 rpm for 10 min at 4 °C was washed with 70% ethanol and resuspended in nuclease-free water.

Intrahepatic WHV DNA RI in Hind*III*-digested total DNA samples was assayed quantitatively against a 2-fold dilution series of linearized pUC-CMVWHV following agarose gel electrophoresis in Tris-acetate (TAE) buffer (lower assay sensitivity, 2 pg WHV DNA/µg cellular DNA). Gels were treated with denaturation solution (Teknova, Hollister, CA) for 10 min and neutralization buffer (Teknova) for 15 min. Following overnight blotting on nitrocellulose membrane (2× SSC), membranes were prehybridized and then hybridized overnight at 60 °C in PerfectHyb Plus Hybridization buffer (Sigma-Aldrich) using a ^{32}P-labeled fragment of purified WHV genome DNA derived from

pUC-CMVWHV. Membranes were then washed extensively (2× SSC ± 1% SDS), airdried, and analyzed by radio imaging.

Intrahepatic WHV cccDNA in Hind*III*-digested total DNA samples was assayed from the singular band in the Southern blot corresponding to the 3.2 kb genome-size DNA species (lower assay sensitivity, 2 pg WHV DNA/μg cellular DNA).

2.2.2. Intrahepatic WHV RNA

For isolating total RNA, frozen liver tissues were homogenized in TRIzol Reagent (Invitrogen) using a plastic pestle. Thereafter, chloroform was added, and samples incubated for 30 min at room temperature. Total RNA within the top layer obtained after centrifugation at 13,000 rpm for 15 min at 4 °C was then extracted with 2-propanol. The pellet obtained after incubation for 5 min at room temperature and subsequent centrifugation at 13,000 rpm for 15 min at 4 °C was washed with 70% ethanol and resuspended in nuclease-free water. The total RNA concentration within a sample was determined with the Nano DropTM 8000 spectrophotometer (Thermo Scientific). Intrahepatic WHV RNA within a sample containing 2 μg of total RNA was assayed quantitatively against a 2-fold dilution series of linearized pUC-CMVWHV following formaldehyde agarose gel electrophoresis in 3-(N-morpholino) propanesulfonic acid (MOPS) buffer (lower assay sensitivity, 2 pg WHV RNA/μg cellular RNA). Gels were treated with denaturation solution (Teknova) for 10 min and neutralization buffer (Teknova) for 15 min. Following overnight blotting on nitrocellulose membrane (2× SSC), membranes were prehybridized and then hybridized overnight at 60 °C in PerfectHyb Plus Hybridization buffer (Sigma-Aldrich) using a ^{32}P-labeled fragment of purified WHV genome DNA derived from pUC-CMVWHV. Membranes were then washed extensively (2× SSC ± 1% SDS), airdried, and analyzed by radio imaging.

2.2.3. Normalization of WHV Nucleic Acids

In all cases, after WHV nucleic acid quantification and image capture, the membranes were stripped with hot wash buffer (10 mM Tris-Cl, pH 7.5–8.0; 1 mM EDTA, 1% SDS) for 10 min at 99 °C, washed extensively in nuclease-free water, and airdried. Membranes were prehybridized and then hybridized overnight at 60 °C in PerfectHyb Plus Hybridization buffer (Sigma-Aldrich) using a ^{32}P-labeled fragment of purified, PCR-amplified woodchuck β-actin as a housekeeping marker. Membranes were washed extensively (2X SSC ± 1% SDS), airdried, and analyzed by radio imaging for subsequent calculations of cellular nucleic acids for normalization of WHV DNA and RNA results.

2.4. Liver IFN Expression

2.4.1. Intrahepatic IFN Transcription

Total RNA was isolated from frozen liver tissues using the RNeasy Mini kit (Qiagen) with on-column digestion by RNase-free DNase I (Qiagen) by following the manufacturer’s protocol. The total RNA concentration within a sample was determined with the Nano DropTM 8000 spectrophotometer (Thermo Scientific). Thereafter, 1 μg of total RNA was reverse transcribed into mRNA with the High Capacity cDNA Reverse Transcription kit (Applied Biosystems) using oligo(dT) by following the manufacturer’s protocol. The cDNA reaction conditions were 10 min at 25 °C, followed by 120 min at 37 °C and 5

min at 85 °C. Triplicate of 5 μL aliquots of cDNA diluted in nuclease-free water were then amplified on a QuantStudio 3 Real Time PCR System instrument. TaqMan Gene Expression Master mix (Applied Biosystems) was used by following the manufacturer's protocol and woodchuck gene-specific primer/probe sets listed in Table S1.

2.4.2. Normalization of IFN Transcription

Separate triplicate of 5 μL aliquots of diluted cDNA were amplified for 18S rRNA as a housekeeping marker using the primer/probe set listed in Table S1. The concentration of each primer was 900 nM, while the concentration of the TaqMan probe was 250 nM. The PCR reaction conditions were 10 min at 95°C, followed by 40 cycles of 15 s at 95°C, and 60 s at 65°C. Woodchuck gene expression was normalized to 18S rRNA expression by using the formula $2^{\Delta Ct}$, where ΔCt indicates the difference in the threshold cycle between 18S rRNA and target gene (i.e., IFN-α, IFN-β, and IFN-γ). For comparison of Ct values between different PCR amplifications, parameters such as threshold (i.e., 0.05) and baseline (i.e., cycles 3–15) were defined and applied to each amplification. Normalized IFN transcription in a given study week was then compared to the normalized IFN expression at the pretreatment baseline at week −1 and the results calculated as a fold-change.

2.5. Liver Histology

Liver biopsy specimens were stored in 10% neutral buffered (phosphate buffer) formalin, embedded in paraffin, sectioned (5 micron), and stained with hematoxylin and eosin for histopathological analysis. Two scoring systems were used. One scale was developed over the years specifically for reading woodchuck liver [68,69], while the other scale was the standard METAVIR scale for reading human liver.

For scoring liver with the woodchuck scale, impressions for separate scores were sought for each portal parameter for the number of portal tracts being scored, including the degree of inflammation and bile duct proliferation in each portal tract. Three to four portal tracs were typically assessed. Lack of portal tract inflammation scored 0, slight inflammation scored 1, additional inflammation without or with slight portal tract distension scored 2, further inflammation with occasional piecemeal necrosis of the limiting plate with some spread of inflammation into the parenchyma scored 3, and more significant portal inflammation with widespread piecemeal necrosis and spread of inflammation into the parenchyma scored 4. The degree of bile duct proliferation was scored similarly as a separate portal parameter (0–4 scale: none, mild, moderate, marked, or severe). Sinusoidal parameters included the degree of parenchymal inflammation (0–4 scale: none, mild, moderate, marked, or severe), the degree of hepatocellular necrosis (0–4 scale: none, mild, moderate, marked, or severe), and the degree of steatosis (0–4 scale: none, mild, moderate, marked, or severe).

For scoring liver with the METAVIR scale, activity scores for portal hepatitis and lobular hepatitis with attending necro-inflammatory changes were considered separately (scale 0–3: none, mild, moderate, or severe), and then the degree of fibrosis was assessed (scale 0–4: none, fibrosis without septa, with few septa, numerous septa without cirrhosis, or cirrhosis). The portal hepatitis score consisted of impressions on the combined degrees of inflammation and tract

expansion, bile duct proliferation, and piecemeal necrosis of the limiting plate with spread of inflammation into the parenchyma. Three to four portal tracs were typically assessed. The lobular hepatitis score consisted of impressions on the combined degree of parenchymal hepatitis, including any contribution from piecemeal necrosis of the limiting plate, prevalence of necrotic hepatocytes, and extent of necrosis (e.g., individual cells to bridging acini). Any presence of steatosis was noted.

Reference

1. Yu, M.; Miller, R.H.; Emerson, S.; Purcell, R.H. A hydrophobic heptad repeat of the core protein of woodchuck hepatitis virus is required for capsid assembly. *J. Virol.* **1996**, *70*, 7085–7091, doi:10.1128/JVI.70.10.7085-7091.1996.
2. Gerin, J.L.; Faust, R.M.; Holland, P.V. Biophysical characterization of the adr subtype of hepatitis B antigen and preparation of anti-r sera in rabbits. *J. Immunol.* **1975**, *115*, 100–105.
